# Functional signatures of de novo *GABBR1* and *GABBR2* variants associated with neurodevelopmental disorders

**DOI:** 10.1038/s41525-026-00558-z

**Published:** 2026-03-09

**Authors:** Michal Stawarski, Noa Bielopolski, Ilana Roitman, Karen Fridman, Shane Wald-Altman, Megan Eitel, Benedict Hui, Anneke Vulto-van Silfhout, Alexander P. A. Stegmann, Adela Chirita-Emandi, Jacqueline Eason, Kirsty Bradshaw, Lewis Darnell, Grażyna Kostrzewa, Rafal Ploski, Romane Meurs, Amandine Batté, Stylianos E. Antonarakis, Martin Gassmann, Bernhard Bettler

**Affiliations:** 1 Department of Biomedicine, University of Basel, Basel, Switzerland; 2 QR Genetics, Tel Aviv, Israel; 3 Valley Children’s Clinic, Renton, WA USA; 4 MultiCare Center for Weight Loss & Wellness, Covington, WA USA; 5 Department of Clinical Genetics, Maastricht UMC+, Maastricht, the Netherlands; 6 Center of Genomic Medicine, University of Medicine and Pharmacy “Victor Babes”, Timisoara, Romania; 7 Regional Center of Medical Genetics Timis, Clinical Emergency Hospital for Children “Louis Turcanu” part of ERN ITHACA, Timisoara, Romania; 8 Department of Clinical Genetics, Nottingham University Hospitals NHS Trust, Nottingham, England; 9 East Midlands Regional Genetics Service, Nottingham University Hospitals NHS Trust, Nottingham, England; 10 Department of Medical Genetics, Warsaw Medical University, Warsaw, Poland; 11 Medigenome, Swiss Institute of Genomic Medicine, Geneva, Switzerland; 12 Department of Genetic Medicine and Development, University of Geneva, Geneva, Switzerland

**Keywords:** Neurodevelopmental disorders, Neurological disorders, Psychiatric disorders, Genetics, Diseases, Molecular medicine

## Abstract

GABA_B_ receptors, the G protein-coupled receptors for the neurotransmitter GABA, are essential for regulating neuronal excitability in the brain. Monoallelic de novo missense variants in *GABBR1* and *GABBR2*, which encode the receptor subunits, have been associated with neurodevelopmental disorders. Here, we investigated the functional impact of seven de novo missense variants in *GABBR1* and *GABBR2* identified in individuals with autism spectrum disorder, intellectual disability, and/or attention deficit/hyperactivity disorder. In vitro functional characterization of these variants revealed a range of gain- and loss-of-function alterations: (i) increased constitutive activity, leading to a corresponding decrease in GABA efficacy; (ii) a significant reduction in GABA potency at the receptor; and (iii) reduced surface expression, resulting in decreased GABA efficacy. While computational predictions indicated pathogenicity for all variants, our study emphasizes the importance of functional studies in clarifying the nature and scope of pharmacological changes—an essential step toward advancing targeted therapies in precision medicine.

## Introduction

GABA_B_ receptors (GBRs) are G protein-coupled receptors for GABA, the main inhibitory neurotransmitter in the central nervous system^1^. They regulate neuronal excitability and synaptic transmission by decreasing cAMP levels and modulating Ca^2+^ and K^+^ channels. GBRs are heterodimers assembled from GB1 and GB2 subunits, which are encoded by the *GABBR1* and *GABBR2* genes, respectively^1,2^. These receptors have evolved quality control mechanisms that prevent unfolded or unassembled subunits from exiting the endoplasmic reticulum (ER) and the Golgi apparatus^2,3^. As a result, GB1 can only reach the cell surface after dimerizing with GB2. Each subunit consists of an extracellular bi-lobed Venus flytrap domain (VFTD), a heptahelical transmembrane domain (TMD), and a C-terminal intracellular domain^4–6^. Within the heterodimeric receptor, GB1 and GB2 perform non-redundant functions. Orthosteric ligands bind to a pocket located within the VFTD of GB1, while the TMD of GB2 is responsible for coupling with the G protein. Receptor activation triggers conformational changes within the heterodimeric structure^6^. The binding of an agonist induces the closure of the VFTD in GB1, bringing the lower lobes of both VFTDs into contact. This alignment is accompanied by a reorientation of the TMDs, which creates a new interface along the transmembrane helix 6 (TM6) of both subunits. Ultimately, these rearrangements lead to the formation of a shallow pocket for G protein binding at the base of the TMD in GB2. Binding of positive allosteric modulators at the TM6 interface stabilizes the active state of the receptor^6–8^.

GBRs are widely expressed in the brain and are found in both glial cells and neurons. In neurons, they play a role at both pre- and postsynaptic sites, where they inhibit neurotransmitter release and contribute to the late phase of inhibitory postsynaptic potentials^1,4^. In microglia, GBRs help to shape inhibitory circuits^9^, while in astrocytes, they facilitate Ca^2+^ increases and influence hippocampal theta and gamma oscillations^10,11^. Pathogenic variants in *GABBR1* and *GABBR2* are associated with a range of symptoms, including neurodevelopmental delays, intellectual disability, autism spectrum disorder (ASD), and, in some cases, epileptic seizures, among others^12–25^. The wide spectrum of symptoms suggests that pathogenic variants in *GABBR1* and *GABBR2* may involve diverse molecular mechanisms, including both gain- and loss-of-function effects, as well as possible adaptive changes. However, only a limited number of reported variants have been evaluated for their effects on GBR function in vitro or in vivo^20–22,24,25^. Advancing our understanding of the disease mechanisms underlying *GABBR1* and *GABBR2* variants will contribute to more accurate diagnosis and, ultimately, the development of targeted treatments.

In this study, we investigated the functional impact of seven de novo missense variants of uncertain significance (VUS) located in *GABBR1* (four variants) and *GABBR2* (three variants). These variants were identified in individuals with delays in motor skills and speech, learning difficulties, intellectual disability, ASD, and/or attention deficit/hyperactivity disorder (ADHD). The seven variants are either absent or rarely observed in the Genome Aggregation Database (gnomAD)^26^ and are classified as deleterious by one or more prediction algorithms. Among these, five caused substantial changes in receptor signaling, while two had smaller yet still significant effects. Based on inheritance patterns, population data, as well as both computational and functional analyses, we classify all seven variants examined as pathogenic or likely pathogenic. Importantly, functional studies reveal both loss-of-function changes and combined gain-of-function and loss-of-function changes of varying degrees that are not predictable through in silico methods and must be considered when developing targeted therapies in precision medicine.

## Results

### In silico analysis of monoallelic de novo *GABBR1* and *GABBR2* variants

**Fig. 1:**
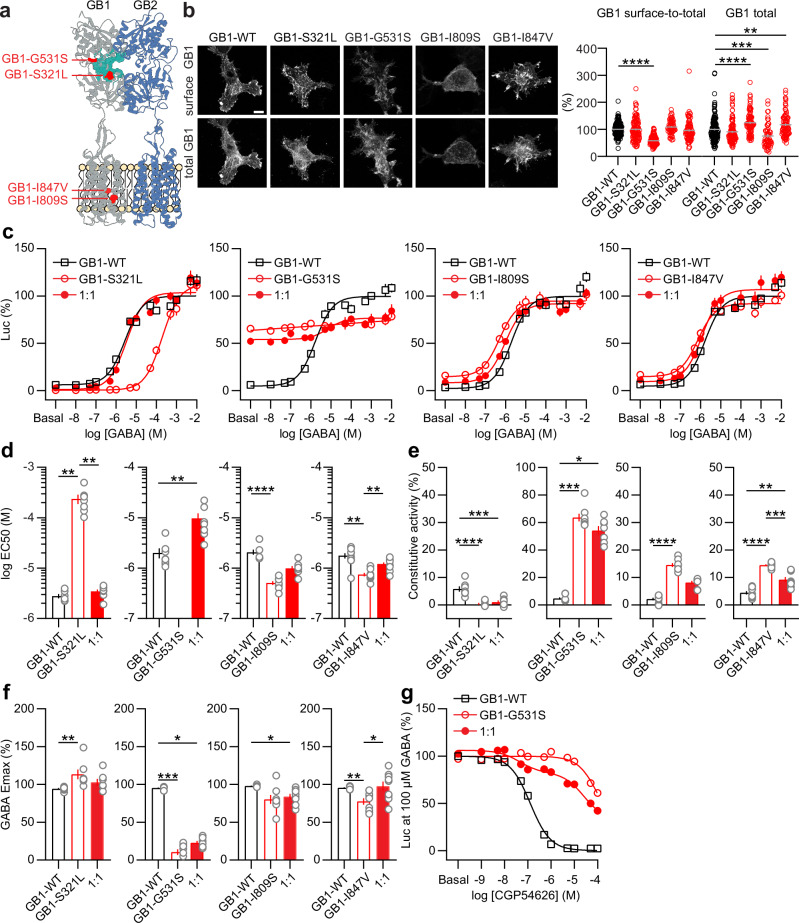
In vitro characterization of GB1 subunit variants. **a** Position of the GB1-S321L, GB1-G531S, GB1-I809S, and GB1-I847V variants in the heterodimeric GBR (PDB: 7C7S). The GB1 (gray) and GB2 (blue) subunits, orthosteric ligand binding pocket in GB1 (cyan), and amino acid changes (red) are indicated. Structural visualizations were performed using the UCSF ChimeraX program^49^. **b** Cell surface and total expression of WT and variant HA-tagged GB1 subunits co-expressed with GB2 in HEK293T cells. GB1 expressed at the cell surface was immunostained with anti-HA antibodies in live cultures. Total GB1 was immunostained after cell permeabilization with anti-GB1 antibodies. Scale bar: 10 μm. Bar graphs show the ratio of surface-to-total GB1 and total GB1 immunofluorescence normalized to GB1-WT. Number of cells analyzed from three independent experiments: GB1-WT, *n* = 151; GB1-S321L, *n* = 112; GB1-G531S, *n* = 93; GB1-I809S, *n* = 58; GB1-I847V, *n* = 83. **c** Dose-response curves of GABA-induced Luc activity from experiments with WT, variant, and WT + variant (1:1 GB1 plasmid ratio) GBRs in transfected cells. Data are normalized to the maximal Luc activity of WT receptors after curve fitting. Dose-response curves were generated using GraphPad Prism. Bar graphs show EC_50_ values (**d**), constitutive activity (**e**), and GABA Emax (**f**) derived from the GABA dose-response curves shown in (**c**). **g** Dose-response curves showing inhibition of GBR activity by the inverse agonist CGP54626. GB1-WT, GB1-G531S, and GB1-WT + GB1-G531S (1:1) receptors were stimulated with 100 µM GABA. Data are normalized to the Luc activity without the inverse agonist. Biphasic decay was used to fit the data for GB1-WT + GB1-G531S receptors, with Hill slopes set to -1 and IC_50_ values constrained by the other two fits. The number of independent experiments and statistical analyses are provided in Supplementary Tables S3 and S4. All data are mean ± SEM, *P < 0.05, **P < 0.01, ***P < 0.001, ****P < 0.0001.

*GABBR1* and *GABBR2* are single-copy genes with no reported paralogues or pseudogenes. We identified seven carriers of monoallelic de novo *GABBR1* or *GABBR2* variants who present with symptoms typically associated with GBR-linked pathologies (Table 1 and Supplementary Table S1). For *GABBR1*, we found four missense variants: (i) NM_001470.4:c.962 C > T p.(Ser321Leu) (hereafter GB1-S321L), (ii) NM_001470.4:c.1591 G > A p.(Gly531Ser)/GB1-G531S, (iii) NM_001470.4:c.2426 T > G p.(Ile809Ser)/GB1-I809S, (iv) NM_001470.4:c.2539 A > G p.(Ile847Val)/GB1-I847V. For *GABBR2*, we detected three missense variants: (i) NM_005458.8:c.493 G > T p.(Asp165Tyr)/GB2-D165Y, (ii) NM_005458.8:c.1289 A > C p.(Gln430Pro)/GB2-Q430P, (iii) NM_005458.8:c.2104 A > G p.(Met702Val)/GB2-M702V. An unrelated individual with a neurodevelopmental disorder carrying the NM_005458.8:c.493 G > T variant has also been reported in ClinVar (Variation ID 1709440)^27^. All variants are either extremely rare or absent in the population database gnomAD v.4.1.0, and are situated at amino acid positions that are highly conserved across vertebrate species (Table 2 and Supplementary Fig. S1). GB1-S321L and GB1-G531S are located within the VFTD, the former at the periphery of the orthosteric ligand binding pocket^28^, and the latter in a buried loop within the hinge region between the two lobes. GB1-I809S and GB1-I847V are located in TM6 and TM7 of the TMD, respectively (Fig. 1a). GB2-D165Y is located at the periphery of the agonist-induced heterodimeric VFTD interface^29^. GB2-Q430P is located in the lower lobe of the GB2 VFTD, while GB2-M702V is situated in the TM6 of the GB2 TMD (Fig. 4a). All variants were scored as pathogenic by at least one prediction tool (CADD^30^, REVEL^31^, SIFT^32^, MutationTaster^33^, PolyPhen-2^34^, ESM1b^35^, and Alphamissense^36^; Table 2). *GABBR1* p.(I847V), which had the lowest CADD score of all variants analyzed (23.9), was only predicted to be pathogenic by MutationTaster. The REVEL score, which is derived from the predictions of 13 in silico tools, indicates pathogenicity (≥0.644) only for the *GABBR1* variants p.(S321L), p.(G531S), and p.(I809S), as well as for the *GABBR2* variant p.(M702V). Of note, while the splice prediction programs AbSplice, SPiP, and Pangolin do not flag the *GABBR1*:c.962 C > T p.(Ser321Leu) variant as splice-defective, the SpliceAI algorithm^37^ predicts a donor site gain 11 nucleotides upstream of the variant, with a delta score of 0.24—slightly above the 0.2 threshold for potentially splice-altering variants. Variants with delta scores below 0.5 are often incompletely penetrant, with a tissue-dependent, variable ratio of normal-to-aberrant transcripts^37^. SpliceVault predicts the most likely aberrant splice transcripts by analyzing population RNA-seq data near splice sites^38^. Based on RNA-seq data from the Genotype-Tissue Expression (GTEx) project, SpliceVault predicts approximately 6% mis-splicing for *GABBR1*:c.962 C > T—including 3.4% exon 8 skipping, which generates an in-frame deletion of 57 amino acids (L265–S321); 2.6% utilization of a cryptic splice site, resulting in a deletion of 4 amino acids (V318–S321); and 0.1% skipping of exons 7 and 8, producing an in-frame deletion of 102 amino acids (C220–S321). All of these predicted transcripts would result in a non-functional GABA-binding domain^6^. While in-frame deletions are unlikely to trigger nonsense-mediated mRNA decay (NMD), the presence of NMD would further support a loss-of-function effect. However, most transcripts harboring *GABBR1*:c.962 C > T are predicted to encode the missense variant p.(Ser321Leu), justifying its functional characterization in our recombinant assay system.

**Table 1:** List of the phenotypic features of individuals with the *GABBR1* and *GABBR2* variants

	Individual 1	Individual 2^50^#	Individual 3	Individual 4	Individual 5	Individual 6	Individual 7
	*GABBR1* NM_001470.4: c.962 C > T p.(Ser321Leu), potential for mis-splicing or NMD	*GABBR1* NM_001470.4: c.1591 G > A p.(Gly531Ser)	*GABBR1* NM_001470.4: c.2426 T > G p.(Ile809Ser)	*GABBR1* NM_001470.4: c.2539 A > G p.(Ile847Val)	*GABBR2* NM_005458.8: c.493 G > T p.(Asp165Tyr)	*GABBR2* NM_005458.8: c.1289 A > C p.(Gln430Pro)	*GABBR2* NM_005458.8: c.2104 A > G p.(Met702Val)
Inheritance	de novo	de novo	de novo	de novo	de novo	de novo	de novo
Gender	M	M	M	F	F	M	F
Age (years/months)	4 y 9 m	12 y	6 y	28 y	7 y 6 m	6 y 5 m	1 y 2 m
Development							
Motor delay	+	*–*	–	+	+	+ dysgraphia	+
Speech delay and speech abnormalities	+	+	+	+	+	+	+
Hypotonia	+	*+*	–	–	+	–	+
Stereotypic movements	+ during sleep	+ recurrent hand flapping	+	+	+	–	–
Epilepsy	+ cryptogenic, localization-related	–	– one episode of absence seizure at 2 y 4 m	–	– multiple focal paroxysmal EEG activities with secondary generalization	–	–
Neuropsychiatric phenotypes							
Intellectual disability/learning difficulties	**+** mild global developmental delay	+ global developmental delay	+	+	+	**–**	**+**
Autism spectrum disorder	**–**	*–*	+	+	**–**	+	*Cannot be diagnosed yet*
ADHD	**–**	*–*	+	**–**	**–**	+	*Cannot be diagnosed yet*
Other	Bicuspid aortic valve and heart murmur, pes planus, bilateral hand and foot differences, macrocephaly	Astigmatism, small scrotum, deep philtrum, exaggerated cupid’s bow, square face, short 4th and 5th toes	Encopresis, tics, behavioral issues, misophonia, mild facial dysmorphism with short filtrum, reverted upper lip, downward palpebral slanting	Tics, high pain threshold, short stature (familial)	Convergent strabismus, abnormal gait, CT unremarkable		Walks with support from both hands from age of 1 y. Points at objects and to ask for water with hand gestures
Pathogenicity (ACMG Standards and Guidelines) ^41^	Pathogenic (PS2-PS3, PM2, PP3)	Pathogenic (PS2-PS3, PM2, PP3)	Likely pathogenic (PS2, PP3, PM2)	Likely pathogenic (PS2, PM2)	Pathogenic (PS2-PS3, PM2, PP3)	Pathogenic (PS2-PS3, PM2, PP3)	Pathogenic (PS2-PS3, PM2, PP3)
Other variants	*NOTCH1* NM_017617.4 c.5281 C > T p.(Arg1761Trp) de novo	–	–	–	*KMT2A* NM_005933.4 c.7435 T > C p.(Cys2479Arg) de novo	–	–

^#^Available in the DECIPHER database (patient #294533).

**Table 2:** Computational prediction of pathogenicity and evolutionary conservation of the *GABBR1* and *GABBR2* variants

	Individual 1	Individual 2	Individual 3	Individual 4	Individual 5	Individual 6	Individual 7
	*GABBR1* NM_001470.4: c.962 C > T p.(Ser321Leu)	*GABBR1* NM_001470.4: c.1591 G > A p.(Gly531Ser)	*GABBR1* NM_001470.4: c.2426 T > G p.(Ile809Ser)	*GABBR1* NM_001470.4: c.2539 A > G p.(Ile847Val)	*GABBR2* NM_005458.8: c.493 G > T p.(Asp165Tyr)	*GABBR2* NM_005458.8: c.1289 A > C p.(Gln430Pro)	*GABBR2* NM_005458.8: c.2104 A > G p.(Met702Val)
Position (GRCh38)	chr6: 29623306 G > A	chr6: 29612590 C > T	chr6: 29605582 A > C	chr6: 29604889 T > C	chr9: 98542010 C > A	chr9: 98406089 T > G	chr9: 98306246 T > C
Frequency in population							
gnomAD v.4.1.0 (frequency)	6.20 × 10^−7^	absent	absent	absent	absent	absent	absent
In silico analysis							
CADD	Deleterious (33)	Deleterious (26.9)	Deleterious (32)	Ambiguous (23.9)	Deleterious (27)	Deleterious (28)	Ambiguous (24.1)
REVEL	Deleterious (0.675)	Deleterious (0.874)	Deleterious (0.897)	Ambiguous (0.507)	Ambiguous (0.495)	Ambiguous (0.537)	Deleterious (0.770)
SIFT	Benign (0.42)	Ambiguous (0.02)	Deleterious (0)	Benign (0.51)	Ambiguous (0.01)	Deleterious (0)	Benign (0.61)
Mutation Taster	Deleterious	Deleterious	Deleterious	Deleterious	Deleterious	Deleterious	Deleterious
PolyPhen-2	Deleterious (0.991)	Deleterious (1.000)	Deleterious (1.000)	Ambiguous (0.864)	Ambiguous (0.976)	Ambiguous (0.745)	Deleterious (0.984)
ESM1b	Benign (-4.77)	Deleterious (-12.66)	Deleterious (-14.56)	Ambiguous (-7.51)	Deleterious (-10.54)	Deleterious (-15.68)	Deleterious (-10.37)
Alphamissense	Deleterious (0.8519)	Deleterious (0.9945)	Deleterious (0.996)	Ambiguous (0.4329)	Deleterious (0.993)	Deleterious (0.998)	Deleterious (0.826)
Conservation							
GERP	4.64	5.86	5.1	5.16	5.39	5.91	5
Conservation in vertebrates	Conserved at the amino acid level	Conserved at the amino acid level	Conserved at the amino acid level	Conserved at the amino acid level	Conserved at the amino acid level	Conserved at the amino acid level	Conserved at the amino acid level

Score thresholds^51^: CADD (range 0–99) benign ≤22.7, deleterious ≥25.3, REVEL (range 0–1) benign ≤0.29, deleterious ≥0.644, SIFT (range 0–1) benign ≥0.08, deleterious ≤0.001, PolyPhen-2 (range 0–1) benign ≤0.113, deleterious ≥0.978, ESM1b benign >−7.5, deleterious <−7.5, Alphamissense (range 0– 1) benign <0.34, deleterious >0.654, Mutation Taster assigns disease causing/polymorphism labels without generating a numerical score, GERP (range −12.3 to +6.17) the higher the score, the more conserved a position.

### Cell surface expression of GB1 subunit variants

We inserted the *GABBR1* and *GABBR2* missense variants into the cDNAs encoding GB1 and GB2 (Supplementary Table S2). GB1 variants with an N-terminal HA-tag were co-expressed with wild-type (WT) GB2 in HEK293T cells. GB1 expressed at the cell surface was labeled in live cultures using anti-HA antibodies while total GB1 was labeled following fixation and permeabilization using monoclonal anti-GB1 antibodies (Fig. 1b). The surface-to-total GB1 ratio was significantly reduced by 50% for GB1-G531S compared to WT, with a concomitant increase in total GB1-G531S expression, which may relate to increased internalization and/or reduced recycling of internalized receptors^3^. The surface-to-total ratios for other variants were not significantly different from WT GB1. However, total expression was significantly reduced for GB1-I809S and increased for GB1-I847V compared to WT GB1 (Fig. 1b).

### Functional characterization of GB1 subunit variants

We functionally characterized GB1 variants using a luciferase-based reporter assay monitoring receptor-mediated G protein activation^20,22,39^. The GABA dose-response curves revealed that the GB1-S321L and GB1-G531S subunits, when co-expressed with WT GB2, exhibited major yet distinct changes in receptor activation compared to WT receptors (Fig. 1c). The EC_50_ of GABA at GB1-S321L receptors was increased approximately 70-fold compared to WT receptors (Fig. 1d). In addition, GB1-S321L receptors showed a small reduction in constitutive activity (Fig. 1e) and a small increase in the GABA Emax (Fig. 1f). In contrast, GB1-G531S receptors exhibited pronounced constitutive activity—approximately 64% of the maximal luciferase signal seen with WT receptors—and essentially showed no response to GABA (Fig. 1c–f). GABA dose-response curves of a mixed population of WT and GB1-S321L receptors, which mimics the monoallelic patient situation, did not differ significantly from those of WT receptors. The GABA dose-response curves for a mixed population of WT and GB1-G531S receptors indicated a gain-of-function effect at GABA concentrations below 1 μM, but a loss-of-function effect at higher concentrations. The GB1-I809S and GB1-I847V receptors exhibited minor yet mostly statistically significant effects on GABA potency (Fig. 1d), constitutive activity (Fig. 1e), and GABA Emax (Fig. 1f). GABA dose-response curves of a mixed population of WT and GB1-I809S or GB1-I847V receptors were similar to those of WT receptors (Fig. 1c–f). Supplementary Table S3 provides a summary of the constitutive activity, EC_50_, and GABA Emax values for the GB1 subunit variants.

### Stabilization of the active conformation of the VFTD by the GB1-G531S subunit

**Fig. 2:**
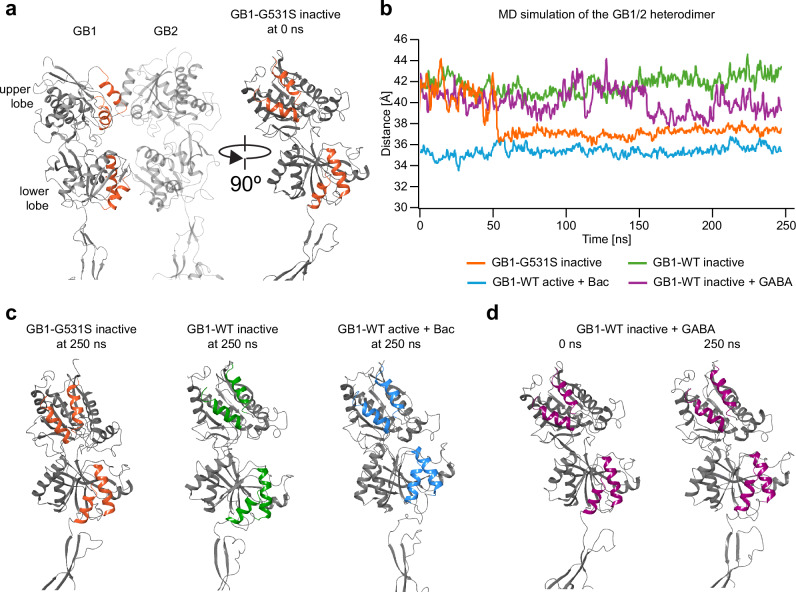
Structural dynamics in MD simulations of the GB1-G531S receptor. **a** Homology modeling of GB1-G531S based on the inactive structure of GBRs. The bi-lobed VFTD structures of GB1 and GB2 are shown (left), along with a 90° rotation of GB1 alone along the vertical axis (right). Homology modeling was performed using the Biologics Suite in Maestro (Schrödinger Release 2022-2). **b** Distance between the upper lobe (center of mass of Cα atoms from residues 222–235 and 247–260) and the lower lobe (center of mass of Cα atoms from residues 347–358 and 368–382) of the GB1 VFTD as a function of time during a 250 ns MD simulation. Graphs were created in Microsoft Excel using data from analyses performed with the MDAnalysis package^48^. **c** VFTD conformation of GB1 at the end of the simulation for GB1-G531S receptors (left) and for WT receptors in the inactive (middle) and active (right, in the presence of baclofen, Bac) conformations. **d** VFTD conformation of GB1 in WT receptors docked with GABA in the inactive conformation (GB1-WT inactive + GABA) at the beginning (0 ns) and end (250 ns) of the simulation. Protein structures in (**c**, **d**) are frames extracted from MD simulations performed using the Desmond MD engine (Schrödinger Release 2022-2).

To test whether the constitutive activity of GB1-G531S receptors is inhibited by an inverse agonist, we generated CGP54626 inhibition curves using the luciferase-based reporter assay. The potency of CGP54626 in inhibiting GB1-G531S receptors in the presence of 100 µM GABA was reduced more than 1000-fold compared to its inhibition of WT receptors (Fig. 1g, IC_50_ values are given in Supplementary Table S4). The GB1-G531S substitution in the hinge region of the VFTD may interfere with stabilization of the inactive conformation by the inverse agonist^29^. To test this hypothesis, we performed molecular dynamics (MD) simulations with a homology model of GB1-G531S receptors (Fig. 2a). The presence of the GB1-G531S substitution caused the VFTD of GB1 to close within the first 50 ns, resulting in a reduction of the distance between the upper and the lower lobe from approximately 41–42 Å to 34–35 Å (Fig. 2a–c). These distances were comparable to those observed with WT receptors in both the inactive and active conformations (Fig. 2b, c), respectively. Docking of GABA into the binding pocket of the VFTD in GB1 of WT receptors in the inactive conformation (inactive + GABA) also decreased the distance between the lobes (Fig. 2b, d), thus validating our MD simulations. The VFTD of the GB1-WT inactive + GABA structure displayed much greater conformational flexibility during the simulation compared to the GB1-G531S receptors. The GB1-G531S substitution, therefore, appears to stabilize the receptor in the active conformation, promoting receptor activity in the absence of GABA. We did not observe the formation of the VFTD and TMD interfaces between GB1 and GB2 that have been reported in the active conformation^6^. Such global structural rearrangements within heterodimeric GBRs might necessitate much longer simulations, similar to what has been observed for metabotropic glutamate receptors^40^.

**Fig. 3:**
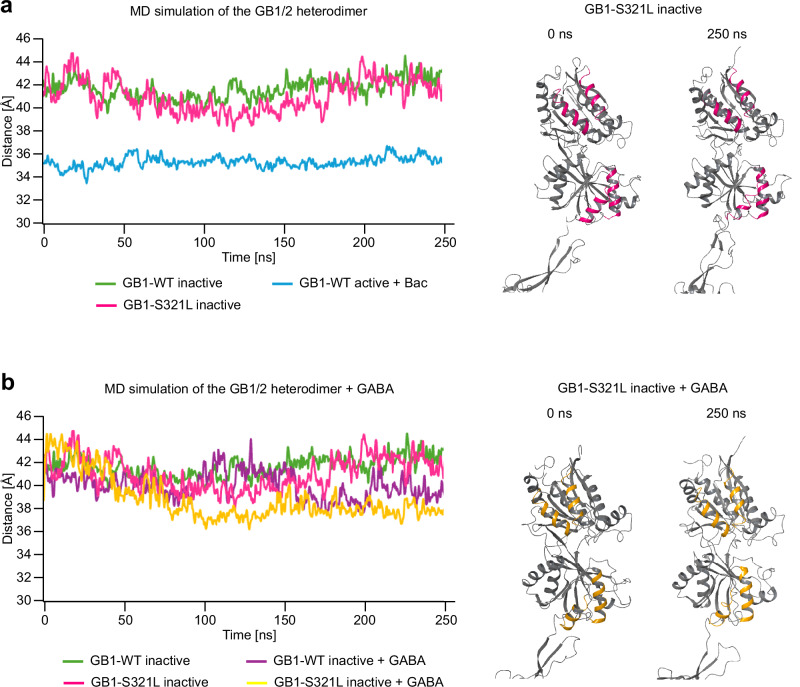
Structural dynamics in MD simulations of the GB1-S321L receptor with and without docked GABA. **a** Distance between the upper and lower lobes of the VFTD in the inactive conformation of GB1-S321L as a function of time during a 250 ns MD simulation (left). For comparison, simulations of the GB1-WT receptor in both inactive and active (in the presence of baclofen, Bac) conformations are shown. VFTD structures of GB1-S321L in the inactive conformation at the beginning (0 ns) and end (250 ns) of the simulation are shown (right). **b** Distance between the upper and lower lobes of the VFTD in GB1-S321L in the inactive conformation in the presence and absence of docked GABA over the 250 ns MD simulation (left). For comparison, corresponding simulations of GB1-WT in the inactive conformation, with and without docked GABA, are shown. VFTD structures of GB1-S321L in the inactive conformation with docked GABA at the beginning (0 ns) and end (250 ns) of the simulation (right). Graphs in (**a**, **b**) were created in Microsoft Excel using data from analyses performed with the MDAnalysis package^48^. Protein structures in (**a**, **b**) are frames extracted from MD simulations performed using the Desmond MD engine (Schrödinger Release 2022-2).

The reduced potency of GABA at GB1-S321L receptors indicates a loss-of-function, whereas the constitutive activity of GB1-G531S receptors reflects a gain-of-function. To directly compare these variants, we performed MD simulations of GB1-S321L receptors in the inactive conformation. In these simulations, the VFTD of GB1-S321L remained in the open, inactive state (Fig. 3a), closely resembling the behavior of GB1-WT receptors and contrasting with the rapid VFTD closure observed in GB1-G531S receptors (Fig. 2b). Docking GABA into the binding pocket of GB1-S321L led to a reduction in the inter-lobe distance of the VFTD—slightly greater than that observed for GB1-WT (Fig. 3b). This finding is consistent with functional data showing that GB1-S321L receptors can still be fully activated by saturating concentrations of GABA, accompanied by a small but significant increase in Emax (Fig. 1c, f). However, GABA potency was markedly reduced at GB1-S321L receptors, with an approximately 70-fold higher EC_50_ compared to GB1-WT (Fig. 1c), indicating a loss-of-function. As expected, this decrease in potency was not captured in the MD simulations, which were initiated with GABA already docked in the binding site.

### Cell surface expression of GB2 subunit variants

**Fig. 4:**
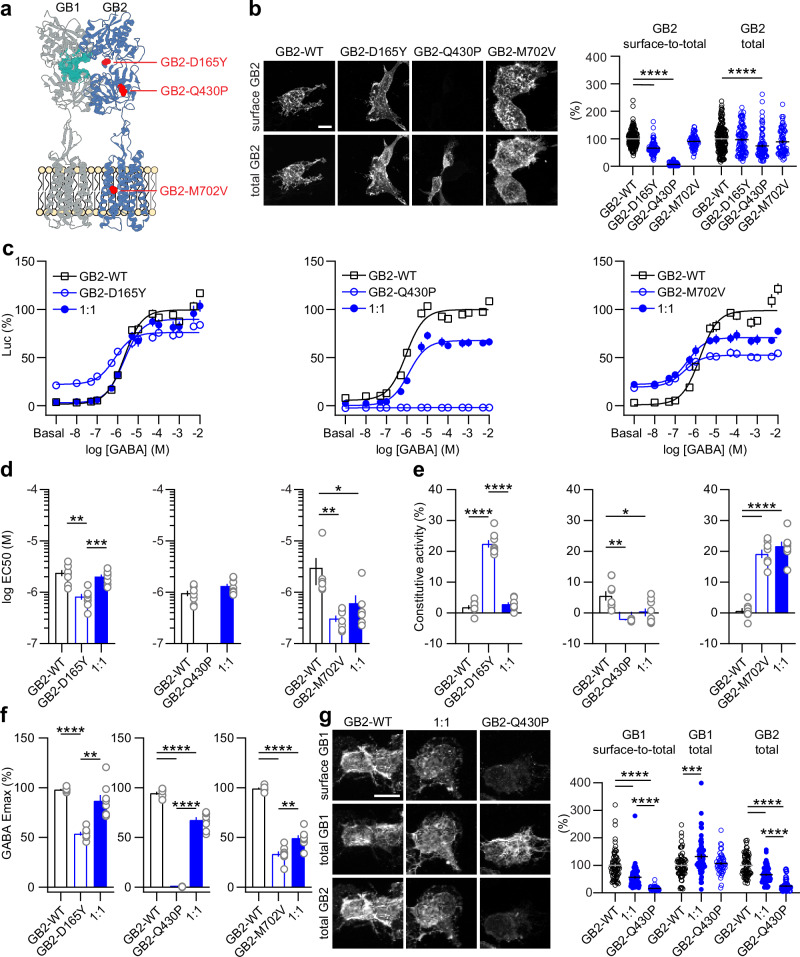
In vitro characterization of GB2 subunit variants. **a** Position of the GB2-D165Y, GB2-Q430P, and GB2-M702V variants in the heterodimeric GBR (PDB: 7C7S). The GB1 (gray) and GB2 (blue) subunits, orthosteric ligand binding pocket in GB1 (cyan), and amino acid changes (red) are indicated. Structural visualizations were performed using the UCSF ChimeraX program^49^. **b** Cell surface and total expression of WT and variant Myc-tagged GB2 subunits co-expressed with GB1 in HEK293T cells. GB2 expressed at the cell surface was immunostained with anti-Myc antibodies in live cultures. Total GB2 was immunostained after cell permeabilization with anti-GB2 antibodies. Scale bar: 10 μm. Bar graphs show the ratio of surface-to-total GB2 and total GB2 immunofluorescence normalized to GB2-WT. Number of cells analyzed from three independent experiments: GB2-WT, *n* = 181; GB2-D165Y, *n* = 100; GB2-Q430P, *n* = 87; GB2-M702V, *n* = 71. **c** Dose-response curves of GABA-induced Luc activity form experiments with WT, variant, and WT + variant (1:1) GBRs in transfected cells. Data are normalized to the maximal Luc activity of WT receptors after curve fitting. Dose-response curves were generated using GraphPad Prism. Bar graphs show EC_50_ values (**d**), constitutive activity (**e**), and GABA Emax (**f**) derived from the GABA dose-response curves shown in (**c**). The number of independent experiments and statistical analyses are provided in Supplementary Table S3. **g** Cell surface and total expression of HA-tagged GB1 co-expressed with GB2-Q430P in HEK293T cells. GB1 expressed at the cell surface was immunostained with anti-HA antibodies in live cultures. Total GB1 and total GB2 were immunostained after cell permeabilization. with anti-GB1 and anti-GB2 antibodies, respectively. Scale bar: 10 μm. Bar graphs show the ratio of surface-to-total GB1, total GB1, and total GB2 immunofluorescence normalized to WT GB1 or GB2 subunits. Number of cells analyzed from three independent experiments: GB2-WT, *n* = 57; GB2-WT + GB2-Q430P (1:1), *n* = 56; GB2-Q430P, *n* = 40. All data are mean ± SEM, *P < 0.05, **P < 0.01, ***P < 0.001, ****P < 0.0001.

GB2 variants containing an N-terminal Myc-tag were co-expressed with WT GB1 in HEK293T cells. In live cultures, anti-Myc antibodies were used to label the GB2 present on the cell surface. Following fixation and permeabilization, total GB2 was labeled using anti-GB2 antibodies (Fig. 4b). The surface-to-total GB2 ratio was significantly reduced for the GB2-D165Y and GB1-Q430P variants (Fig. 4b). Notably, the GB2-Q430P variant was predominantly retained inside the cell and exhibited lower total protein levels compared to WT. In contrast, the GB2-M702V variant was normally expressed at the cell surface and demonstrated WT total protein levels.

### Functional characterization of GB2 subunit variants

GABA dose-response curves revealed that the GB2-D165Y and GB2-M702V, when co-expressed with WT GB1, exhibited significantly increased constitutive activity along with a corresponding reduction in GABA Emax. Additionally, both variants displayed a slight decrease in EC_50_ for GABA (Fig. 4c–f). Notably, the effects observed for GB2-M702V, but not for GB2-D165Y, were also present in a mixed population of variant and WT receptors. The GB2-Q430P variant, due to loss of cell surface expression, was found to be functionally inactive. Mixed populations of WT and GB2-Q430P receptors exhibited a significantly reduced GABA Emax, supporting a reduction in the number of functional receptors expressed at the cell surface (Fig. 4c–f). Supporting this hypothesis, the GB2-Q430P subunit significantly decreased the surface-to-total ratio of GB1 when co-expressed with WT GB2 (Fig. 4g). As a control, GB2-Q430P alone failed to promote the surface expression of GB1. Supplementary Table S3 provides a summary of constitutive activity, EC_50_, and GABA Emax values for the GB2 subunit variants.

## Discussion

Genomic sequencing has enabled the identification of variants in *GABBR1* and *GABBR2* in individuals with neurodevelopmental delays, intellectual disability, epilepsy, autism, and other neurological and psychiatric conditions^12–25^. Although many of these variants are computationally predicted to be pathogenic, only a limited number have directly been shown to affect receptor function^20–22,24,25^. In this study, we characterized four novel variants in *GABBR1* and three in *GABBR2* that were identified in individuals with neurodevelopmental disorders. Six of the seven variants were predicted to be deleterious by the widely used CADD and/or REVEL scores, with *GABBR1* p.(I847V) being the only variant classified as ambiguous by both. However, *GABBR1* p.(I847V) is still predicted to be deleterious by Mutation Taster. Our experimental findings demonstrate that all variants significantly alter receptor function in vitro, with *GABBR1* p.(I809S) and p.(I847V) showing smaller but significant alterations. Based on inheritance patterns, population data, and available computational and in vitro functional evidence, we classify all seven variants as pathogenic or likely pathogenic according to ACMG criteria^41^ (Table 1).

The GB1-G531S, GB1-I809S, and GB1-I847V variants characterized in this study are the first *GABBR1* variants reported to significantly increase constitutive activity. The GB1-G531S variant, located in the hinge region between the two lobes of the VFTD, exhibits particularly high constitutive activity, reaching 64% of the maximal activity observed in the WT receptor. The GB1-I809S and GB1-I847V variants display more moderate increases in constitutive activity, with increases of 14% and 15%, respectively. Interestingly, despite variability in age among affected individuals, both GB1-I809S and GB1-I847V lead to clinical symptoms similar to those associated with GB1-G531S. These symptoms include speech delay, speech abnormalities, stereotypic movements, and intellectual disability (Table 1). Individuals with the GB1-G531S variant also exhibit hypotonia, while carriers of GB1-I809S and GB1-I847V are additionally diagnosed with ASD. Overall, the severity of clinical symptoms does not appear to directly correlate with the extent of constitutive activity. Notably, due to their elevated baseline activity in the absence of GABA, constitutively active variants exhibit a corresponding reduction in GABA efficacy. Moreover, experiments with *Gabbr2*^I704N/+^ mice, which express a constitutively active epileptic encephalopathy variant, demonstrated that strong constitutive GBR activity induces an adaptive downregulation of the receptor and its associated signaling components, including G protein subunits^42^. This compensatory downregulation mitigates the effects of constitutive activity and results in a combination of gain-of-function and loss-of-function effects. Similarly, the GB1-G531S variant analyzed in this study may trigger a comparable adaptive downregulation of GBR signaling. Experiments with *Gabbr2*^I704N/+^ mice underscore the importance of examining pathogenic *GABBR1* and *GABBR2* variants in the intact brain at physiological expression levels. In contrast, experiments in transfected neurons failed to reveal the dual gain-of-function and loss-of-function effects observed in vivo—namely, increased constitutive inhibition during basal network activity and decreased receptor-mediated inhibition during sensory-stimulated activity^25^. Our MD simulations indicate that the GB1-G531S subunit promotes closure of the GB1 VFTD, thereby stabilizing the receptor’s active state^43^. The GB1-I809S and GB1-I847V variants, located in TM6 and TM7, respectively, and the GB2-M702V variant in TM6, likely increase constitutive activity by promoting the TM5/6-TM6/7 interface, which forms between GB1 and GB2 to stabilize the receptor’s active state^6,8^. GABA displays significantly reduced potency at the GB1-S321L variant, consistent with a loss-of-function phenotype. The *GABBR1*:c.962 C > T variant that encodes GB1-S321L may to some extent also cause skipping of exon 7 and/or exon 8, or lead to other forms of aberrant splicing. Such splicing defects would similarly result in a loss-of-function phenotype by producing non-functional receptors. The GB2-D165Y variant is situated within the agonist-induced interface between the VFTDs of GB1 and GB2, suggesting it may trigger constitutive activity by stabilizing inter-subunit interactions in the VFTDs when in the active state^6^. We can only speculate on the reasons why some variant receptors exhibit reduced surface expression. The constitutive activity of GB1-G531S and GB2-D165Y may potentially enhance receptor internalization, similar to what has been reported for agonist-induced internalization^44^. In contrast, the GB2-Q430P variant, which is essentially absent from the cell surface and exhibits reduced overall protein levels, may be more prone to degradation due to misfolding and failure to exit the ER^3^. The individual carrying the GB2-Q430P variant presents relatively mild symptoms. Interestingly, this variant shows functional and symptomatic equivalence to the previously reported GB1-G673D variant^22^. These observations indicate that loss-of-function variants may be better tolerated than hypo- or hypermorphic variants. One plausible explanation is that, when non-functional receptors fail to reach or remain at the plasma membrane, they do not compete with WT receptors for space at the membrane, allowing the WT receptors to compensate for the lost activity.

While computational predictions are generally valuable for assessing the potential pathogenicity of variants, functional studies are essential for determining whether variants result in gain- or loss-of-function effects and for assessing the extent of these functional changes. For example, although *GABBR1* p.(I809S) is predicted to be deleterious by CADD, REVEL, and other computational algorithms, it exhibits only relatively small functional alterations. Our study also demonstrates that MD simulations can serve as valuable tools for identifying functional changes and molecular mechanisms associated with specific pathogenic variants, even in the absence of wet-lab experiments, as previously suggested^20^. Overall, our findings indicate that the functional changes observed with pathogenic variants in *GABBR1* and *GABBR2* can be broadly categorized into three types: (1) reduced GABA potency at the receptor, (2) increased constitutive receptor activity, and (3) decreased receptor surface expression. As discussed above, some variants exhibit both increased constitutive activity and reduced surface expression. Understanding the type of functional alteration is crucial for developing targeted therapies. Reduced GABA potency and decreased surface expression result in a loss-of-function, which may be treatable with positive allosteric modulators that enhance GABA activity at the WT receptor in monoallelic individuals. Several *GABBR1* and *GABBR2* variants result in increased constitutive activity, which concomitantly reduces the GABA Emax, as the receptor is partially active in the absence of GABA. Interestingly, these constitutively active receptors also show a decrease in EC_50_ for GABA, a phenomenon seen in other constitutively active GPCRs^45^. The in vivo effects of such constitutively active variants are likely to be complex. At low GABA levels—such as during ambient GABA concentrations—constitutive activity and enhanced GABA potency may lead to a gain-of-function. Conversely, at high GABA concentrations during synaptic activity, the reduced efficacy of GABA could result in a loss-of-function. Although an inverse agonist targeting GBRs could reduce their constitutive activity, this strategy risks further diminishing GABA-mediated signaling during synaptic activity. Notably, the clinical features associated with variants exhibiting increased constitutive activity (e.g., GB1-G513S, GB1-I809S, GB1-I847V, GB2-DY165Y, GB2-M702V)—a gain-of-function—overlap with those observed in variants characterized by reduced GABA potency (e.g., GB1-S321L) or impaired surface expression (e.g., GB2-Q430P)—a loss-of-function. As discussed above, this clinical overlap is partly explained by a recent study in the *Gabbr2*^I704N/+^ mouse model, which demonstrates that elevated constitutive receptor activity can induce compensatory downregulation of the receptor and its signaling components, ultimately attenuating the maximal response to synaptically released GABA and producing mixed gain-of-function and loss-of-function effects on network activity in the brain^42^. To clearly establish a direct causal link between monoallelic *GABBR1* or *GABBR2* variants and neuronal dysfunction or disease phenotypes, it will therefore be essential to generate mouse models in which the variant is introduced into the endogenous gene locus. Such models preserve the physiological expression levels of both WT and variant alleles within their native neuronal context, enabling detailed analyses of synaptic and network function through both in vitro and in vivo electrophysiology, as well as comprehensive biochemical profiling. These animal models will also inform therapeutic strategies. For example, the *Gabbr2*^I704N/+^ mouse model indicates that strongly constitutively active receptor variants are best targeted with PAMs exhibiting minimal ago-PAM activity, in order to prevent further adaptive downregulation of receptor signaling^42^.

A substantial number of *GABBR1* and *GABBR2* variants listed in ClinVar are found in individuals exhibiting phenotypes typically linked to GBR-related disorders. However, due to the lack of functional validation, many of these variants are classified as variants of uncertain significance (VUS), despite receiving high pathogenicity scores from in silico prediction tools. As shown in this study, functional validation using recombinant assay systems offers a rapid and cost-effective approach to assess the impact of such VUS on GBR function. These assays can distinguish between loss-of-function and gain-of-function effects, thereby enabling the establishment of mechanistic links between receptor dysfunction and specific disease phenotypes, which can subsequently be validated in animal models. Such insights are critical for the development of targeted therapies in the context of precision medicine.

## Methods

### Individuals

Each individual was evaluated at their respective clinical institution, where written informed consent for publication was obtained from their legal guardians. All relevant ethical regulations, including the Declaration of Helsinki, were followed. The Deciphering Developmental Disorders (DDD) study, which identified the *GABBR1* p.(G531S) variant, has UK Research Ethics Committee (REC) approval (10/H0305/83, granted by the Cambridge South REC, and GEN/284/12 granted by the Republic of Ireland REC). de novo occurrence of variants was confirmed using Sanger sequencing when they did not meet next-generation sequencing (NGS) quality metrics for a true positive call (Supplementary Table S1).

### Genomic DNA sequencing

Individual 1, *GABBR1* c.962 C > T p.(S321L): Whole exome sequencing (WES) was performed on genomic DNA using the Human Comprehensive Exome kit from Twist Bioscience to enrich the whole exome (Supplementary Fig. S2). The exome was sequenced using an Illumina sequencing system with paired-end reads at a minimum coverage of 20× of 95% of the target regions. This individual’s exome DNA sequences were aligned to the human reference genome (build UCSC hg19) with BWA-mem. Variants were called using GATK, and QC was performed as part of an in-house developed pipeline based on GATK (Sentieon) best practices. Variants identified by the GATK-based bioinformatics pipeline were uploaded to the Fabric Genomics Analysis platform, which was used to annotate, analyze, and classify these identified variants. Sanger confirmation was performed on the reported variant.

Individual 2, *GABBR1* c.1591 G > A p.(G531S): The variant was identified as part of the Deciphering Developmental Disorders (DDD) study^46^. De novo occurrence was confirmed using bi-directional Sanger sequencing (Supplementary Fig. S3).

Individuals 3 and 7, *GABBR1* c.2426 T > G p.(I809S) and *GABBR2* c.2104 A > G p.(M702V): WES was performed using Illumina’s sequencing-by-synthesis method with paired-end reads (Supplementary Figs. S4 and S5). Sequence reads for each sample were mapped to the human reference genome (GRCh37/hg19) using Burrows-Wheeler Aligner (BWA-MEM) software. Variant calling was conducted using GATK algorithms for nuclear DNA. Variant data were annotated using VcfAnno and VEP, utilizing information from gnomAD, ClinVar, and HGMD. The sequencing run included in-process reference samples for quality control, which met established thresholds for sensitivity and specificity.

Individual 4, *GABBR1* c.2539 A > G p.(I847V): Genome sequencing (short-read) was performed using an Illumina NovaSeq 6000 after processing genomic DNA with the Illumina DNA PCR-Free Prep Kit in the Laboratory for Genome Diagnostics at Radboudumc in Nijmegen. Read alignment and variant calling were conducted using the DRAGEN Bio-IT Platform (Illumina). Variants were annotated with an in-house developed pipeline. De novo occurrence of the reported variant was confirmed using bi-directional Sanger sequencing (Supplementary Fig. S6).

Individual 5, *GABBR2* c.493 G > T p.(D165Y): Whole exome sequencing was performed at the Department of Medical Genetics at the Medical University of Warsaw. The exome from the proband was captured using the SureSelectXT Human All Exon v7 kit (Agilent) and sequenced on the Illumina HiSeq 1500 platform, achieving coverage of over 20× for 94% of the target sequences. Alignment was performed against the human genome assembly GRCh38. The pathogenicity of all protein-altering variants was evaluated using the GnomAD database (release v.3.0). De novo occurrence of the reported variant was confirmed using amplicon deep sequencing with the Illumina Nextera XT kit (Supplementary Fig. S7). Of note, an unrelated individual with this variant is reported in ClinVar (Variation ID: 1709440).

Individual 6, *GABBR2* c.1289 A > C p.(Q430P): DNA was enriched for the complete coding regions and splice site junctions of most genes in the human genome using a proprietary capture system developed by GeneDx for next-generation sequencing with copy number variation (CNV) calling (NGS-CNV). The enriched targets were simultaneously sequenced with paired-end reads on an Illumina platform. Bi-directional sequence reads were assembled and aligned to reference sequences based on NCBI RefSeq transcripts and the human genome build GRCh37/UCSC hg19. The de novo occurrence of the reported variant was confirmed using bi-directional Sanger sequencing (Supplementary Fig. S8).

### Plasmid and reagents

Plasmids encoding human HA-GB1b, Myc-GB2, and SRE-FLuc were as described earlier^22,47^. GB1 and GB2 mutations were generated with the Q5 Site-Directed mutagenesis kit (New England Biolabs, Ipswich, USA) with primers designed in NEBaseChanger (New England Biolabs) and purchased from Microsynth AG (Balgach, Switzerland). Primer sequences are provided in Supplementary Table S2. DNA was PCR-amplified with the Phusion Hot Start II Polymerase (Thermo Fisher Scientific, Waltham, USA). Anti-HA antibody (#11867423001; RRID AB_390918) was from Roche (Basel, Switzerland), anti-Myc (#ab9106; RRID AB_307014) and anti-GB1 (#55051; RRID AB_941703) were from Abcam (Cambridge, UK), anti-βactin (#4970; RRID AB_2223172) was from Cell Signaling Technology (Danvers, USA), anti-GB2 (#322205; RRID AB_2620061) was from Synaptic Systems (Göttingen, Germany), anti-rat AF488 (#A-21208; RRID AB_2535794), anti-rabbit AF488 (#A-11008; RRID AB_143165), and anti-mouse AF647 (#A-31571; RRID:AB_162542) were from Thermo Fisher Scientific (Waltham, USA), anti-guinea pig DyLight405 (#706475148; RRID AB_2340470) was from Jackson ImmunoResearch (West Grove, USA). GABA (#0344) and CGP54626 (#1088) were from Tocris Bioscience (Bristol, England), poly-L-Lysine (P1399) was from Sigma (Burlington, USA), and Lipofectamine 2000 was from Thermo Fisher Scientific (Waltham, USA).

### Receptor surface expression

For immunocytochemistry of HA- and Myc-tagged GB1 and GB2 subunits expressed at the cell surface, cultured cells were cooled for 5 min and incubated with anti-HA and anti-Myc primary antibodies (1:1000, 15 min at 4 °C), followed by Alexa Fluor 488-conjugated secondary antibodies (1:500, 90 min at RT). After fixation and permeabilization, total expression of the GB1 or GB2 subunits was assessed using primary antibodies specific to these subunits (1:1000; overnight at 4 °C) and Alexa Fluor 647-conjugated secondary antibodies (1:500, 90 min at RT). In the experiment examining the effect of GB2-Q430P on GB1 surface trafficking, permeabilized cells were simultaneously immunostained for GB1 and GB2, followed by Alexa Fluor 647-conjugated and DyLight405-conjugated secondary antibodies (1:500). Images were acquired using a Zeiss LSM880 laser scanning confocal microscope fitted with a PLAN APO 63× objective. The data were processed using Fiji software, and fluorescence intensity was quantified in maximum intensity projection images within regions of interest (ROIs) defined around individual cells, with background adjustments applied. The calculated ratios were normalized to the levels of WT receptors.

### Functional analysis

Receptor-mediated G protein activation was assessed in HEK293T cells stably expressing Gαqi and transiently transfected with GB1, GB2, and the luciferase reporter SRE-Fluc, which monitors PLC activity^22^. The plasmids for GB1 and GB2 were transfected at a 1:1 ratio. In co-expression experiments with WT and variant subunits, half of the variant plasmid DNA was replaced with WT plasmid DNA. GBRs were activated with GABA for 6 h. FLuc activity in lysed cells was measured using the Dual-Luciferase Assay Kit (Promega, Madison, USA) and a Tecan Spark microplate reader. Luminescence signals were adjusted by subtracting the background luminescence measured in cells transfected solely with the SRE-FLuc reporter. The data were then analyzed using single exponential dose-response models or linear models for GB1-G531S and GB2-Q430P, and normalized to the maximum luminescence (top plateau) observed with WT receptors. Constitutive activity, defined as luminescence measured in the absence of GABA, corresponds to the bottom plateau of the fits. The GABA Emax was obtained by subtracting the constitutive activity from the top plateau. All values are presented as a percentage of the maximum luminescence observed with WT receptors.

### Molecular dynamics (MD) simulations

Inactive and active structures of the receptor (PDB: 7C7S, 7EB2) were prepared for MD simulations as described^20^. Baclofen was retained in the active structures (PDB: 7EB2), while antagonists were removed from the inactive structures. Homology modeling was performed using the Biologics Suite in Maestro (Schrödinger Release 2022-2, Schrödinger, LLC, New York, NY). H-bond network optimization was carried out assuming a neutral pH of the solution, followed by water molecules removal. An all-atom minimization step was carried out to remove unfavorable steric clashes until a convergence was reached or with a maximum RMSD of 0.3 Å from the original conformation using force field module OPLS4e. No steric clashes were reported after the final minimization step. For MD simulations, systems were built for WT, GB1-G531S, and GB1-S321L receptors using the system builder panel of Desmond (Schrödinger Release 2022-2). The SPC solvent model was used, and the force field was set to OPLS4e. Membrane was placed using OPM (https://opm.phar.umich.edu/). The protein was inserted into an orthorhombic box with buffer dimensions 10 × 10 × 10 Å^3^ and NPγT ensemble. The total simulation time for each system was set at 250 ns. Simulations were set to run at 300.0 K and at 1.01325 bar. The option to relax model systems before simulations was selected. Trajectories were analyzed with the MDAnalysis package^48^. Euclidian distance between the upper and lower lobe of the VFTD in GB1 was calculated from Gaussian-filtered Cα center of mass coordinates (upper lobe: aa222–235 and aa247–260; lower lobe: aa347–358 and aa368–382) extracted from each frame of the simulation.

### Statistical analysis

Statistical analysis was with Prism 10 from GraphPad (La Jolla, USA). Data were tested for normality using the Shapiro–Wilk test and for homoscedasticity with the Bartlett’s test, followed by ordinary one-way ANOVA, Welch’s ANOVA or Kruskal-Wallis tests and appropriate post-hoc tests. Outliers were not removed from the analysis. Data are presented as mean ± SEM. Statistical significance threshold was set at *P* < 0.05. Exact P-values and statistical tests used for each comparison are listed in Supplementary Tables S3 and S4.

## Supplementary information

Stawarski et al. NPJ Genomic Medicine_Supplementary Information Revision

## Data Availability

The raw data supporting the conclusions of this article will be made available by the authors, without undue reservation. The variants analyzed will be submitted to the ClinVar database (https://www.ncbi.nlm.nih.gov/clinvar).
