# Supplementary material for: Functional signatures of de novo GABBR1 and GABBR2 variants associated with neurodevelopmental disorders
Source: NPJ Genom Med. 2026 Mar 9;11:23. doi: 10.1038/s41525-026-00558-z (PMC13103378; doi:10.1038/s41525-026-00558-z)

Clinical description of individuals with *GABBR1* and *GABBR2* variants

Individual 1, *GABBR1* c.962C>T p.(S321L): Contact initiated by parents. 2-year-old male with cryptogenic localization-related epilepsy, hypotonia, bicuspid aortic valve and heart murmur, mild global developmental delay, pes planus, bilateral hand and foot differences, macrocephaly, and unique physical characteristics.

Individual 2, *GABBR1* c.1591G>A p.(G531S): Contact initiated by Jacqueline Eason, Kirsty Bradshaw, and Lewis Darnell. A 12-year-old male with global developmental delay. Initial milestones were unremarkable, with sitting achieved at 6 months; however, significant developmental delay became evident by age 2. He began walking in his 3rd year, though mobility was hindered by hypotonia and tight Achilles tendons, necessitating bilateral tenotomy and subsequent bilateral gastrocnemius lengthening. At age 12, he remains hypotonic, can walk short distances, but requires a wheelchair for longer distances due to fatigue. He can write and read his first name, attends a school for special educational needs, and requires assistance with daily activities such as washing. He is generally happy, speaks in short sentences, and exhibits hand stereotypies, including recurrent hand flapping. Growth parameters are within normal limits, with height on the 79th percentile and OFC on the 36th percentile. He has significant myopia, a square face, full lips with a prominent Cupid's bow, and brachycephaly. Additional features include pes planus and a wide-based gait.

Individual 3, *GABBR1* c.2426T>G p.(I809S): Contact initiated by Adela Chirita-Emandi. A 6-year-old male with normal perinatal and motor development, but with moderate intellectual disability and delayed language acquisition (began speaking at 3 years and 6 months). He presents with tics, behavioral issues, misophonia, and lacks sphincter control. Some behaviors are consistent with autism spectrum disorder and obsessive-compulsive traits. He experienced one episode of absence seizure at 2 years and 4 months but has not received antiepileptic medication. Mild facial dysmorphism, including a short philtrum, everted upper lip, and downward slanting palpebral fissures. No family history of similar conditions is reported.

Individual 4, *GABBR1* c.2539A>G p.(I847V): Contact initiated by Anneke Vulto-van Silfhout. A 28-year-old female born at 38 weeks of gestation via cesarean section due to maternal factors. She required tube feeding briefly after birth, but feeding normalized afterward. Due to developmental delay, she attended medical day care from age 2, with no signs of regression. She has a severe intellectual disability with a disharmonic developmental profile, achieving a social independence level of 2 years and 3 months on the Vineland Adaptive Behavior Scale. She is also diagnosed with autism spectrum disorder and sensory processing disorder. She

experiences eye tics, investigated by EEG with no signs of epilepsy, and has a habit of rubbing her left eye, though ophthalmologic and cerebral MRI findings are normal. Her hearing is normal, and she has a high pain threshold. Height of 143 cm (-4 SD), weight of 55.1 kg (+1.5 SD), and head circumference of 56.1 cm (+0.5 SD). Distinctive facial features include prominent eyebrows, deep-set eyes, a full nasal tip, and full lips. Mother with short stature (148 cm, -3.5 SD) and a brother with autism spectrum disorder and high IQ.

Individual 5, *GABBR2* c.493G>T p.(D165Y): Contact initiated by parents. A 7-year-old female with neurodevelopmental delay, intellectual disability, and motor abnormalities, including an abnormal gait, hypotonia, and reduced motor coordination. She was born at 41 weeks of gestation with a birth weight of 3700 g and an APGAR score of 10. Milestones were significantly delayed: she could sit unassisted at 1.5 years, stand independently at 2.5 years, and walk unassisted at age 5. Currently non-verbal, she displays notable behavioral abnormalities, including escalating self-injurious behaviors and emotional volatility. EEG and CT scans were normal until age 3, at which point EEG revealed multiple localized seizures. She also has periodic convergent strabismus in her right eye.

Individual 6, *GABBR2* c.1289A>C p.(Q430P): Contact initiated by Benedict Hui and Megan Wells. A 6-year-old male diagnosed with Autism Spectrum Disorder, Attention-Deficit/Hyperactivity Disorder, and Dysgraphia. Born at 39 weeks via C-section following an uncomplicated pregnancy, with a birth weight of 3700 g and an Apgar score of 9. Developmental milestones were achieved within normal limits: sitting at 6 months, first words at 6 months, and walking at 11 months. The patient is verbal and shows advanced abilities in reading, spelling, and computational mathematics. However, he exhibits social-emotional delays and difficulties with expressive and receptive communication commonly associated with ASD. Impulse control challenges and fine motor delays are present, though gross motor skills are intact. There is no history of seizures.

Individual 7, *GABBR2* c.2104A>G p.(M702V): Contact initiated by Adela Chirita-Emandi. A 14-month-old female born after an uneventful pregnancy (G2P2, birth weight 3400 g) with an APGAR score of 4/7, requiring resuscitation at birth. She presents with global developmental delay, hypotonia, and delays in both fine and gross motor skills. Physical therapy began at 6 months. She does not walk independently but walks with crossed feet when supported by both hands. Growth parameters are within normal limits for age, with no dysmorphic facial features. Patellar reflexes are present. She experiences mild sleep disturbances and has

hepatosplenomegaly, though liver enzymes remain within normal ranges. Brain ultrasound findings are unremarkable. No family history of similar conditions is reported.

Supplementary Table S1. Sequence validation of *GABBR1* and *GABBR2* variants

Variant	Coverage	Validation	Segregation
***GABBR1* p.(S321L)**	WES, >20x	Sanger	Proband: Het<br>Mother: WT<br>Father: WT
***GABBR1* p.(G531S)**	WGS, 47x	Sanger	Proband: Het<br>Mother: WT<br>Father: WT
***GABBR1* p.(I809S)**	WES, 176x	NGS quality metrics for a true positive call met	Proband: Het<br>Mother: WT<br>Father: WT
***GABBR1* p.(I847V)**	WGS, 45x	Sanger	Proband: Het<br>Mother: WT<br>Father: WT
***GABBR2* p.(D165Y)**	WES, >20x	Amplicon deep sequencing	Proband: Het<br>Mother: WT<br>Father: WT<br>Brother: WT
***GABBR2* p.(Q430P)**	CES, 124x	Sanger	Proband: Het<br>Mother: WT<br>Father: WT
***GABBR2* p.(M702V)**	WES, 86x	NGS quality metrics for a true positive call met	Proband: Het<br>Mother: WT<br>Father: WT

WES, whole exome sequencing; WGS, whole genome sequencing; CES, clinical exome sequencing; NGS, next generation sequencing.

Supplementary Table S2. Sequences of mutagenesis primers (lowercase indicates the mutations)

Variant	Primer name	Sequence
***GABBR1* p.(S321L)**	GABBR1p.(S321L)_FWD	GTCTTCACTTtGACTCTGGAC
	GABBR1p.(S321L)_REV	CTCAGTGGTCTGCTG
***GABBR1* p.(G531S)**	GABBR1p.(G531S)_FWD	TGATGCCAGCaGCTCTCGGAT
	GABBR1p.(G531S)_REV	AACACCACATGGCCAG
***GABBR1* p.(I809S)**	GABBR1p.(I809S)_FWD	GGCATGGCTAgCTACAATGTG
	GABBR1p.(I809S)_REV	CACAGCCCGGTGATC
***GABBR1* p.(I847V)**	GABBR1p.(I847V)_FWD	CTCCTCCTATgTCACTCTTGTTG
	GABBR1p.(I847V)_REV	AAAACTATGGCAAGAGAG
***GABBR2* p.(D165Y)**	GABBR2p.(D165Y)_FWD	TGTTCTAGCCtatAAGAAAAAATACCCTTATTTC
	GABBR2p.(D165Y)_REV	GGCGTGGTTGCAGCA
***GABBR2* p.(Q430P)**	GABBR2p.(Q430P)_FWD	AAATTTACTCcATTTCAAGACAGCAG
	GABBR2p.(Q430P)_REV	AATGGTCCCCATTCTCTC
***GABBR2* p.(M702V)**	GABBR2p.(M702V)_FWD	CGTGGGGATCgTGTGCATCAT
	GABBR2p.(M702V)_REV	TTGTAGACACTCATCCCG

Supplementary Table S3. Fit parameters for GABA dose-response curves

GABBR1	WT (n=7)	p.(S321L) (n=7)	1:1 (n=7)	Statistics	WT& p.(S321L)	WT&1:1	p.(S321L)&1:1
**Constitutive activity (%)**	5.80	0.43	1.05	ANOVA (P<0.0001) with Tukey's post-hoc	P<0.0001	P=0.0003	P=0.7948
**Emax (%)**	94.20	113.40	102.80	Kruskal-Wallis (P=0.0048) with Dunn's post-hoc	P=0.0067	P=0.2547	P=0.5454
**EC_{50} [µM]**	2.79	237.30	3.46	Welch's ANOVA (P=0.0017) with Dunnett's T3 post-hoc	P=0.0090	P=0.3263	P=0.0092
GABBR1	**WT (n=8)**	**p.(G531S) (n=8)**	**1:1 (n=7)**	**Statistics**	**WT& p.(G531S)**	**WT&1:1**	**p.(G531S)&1:1**
**Constitutive activity (%)**	4.75	63.58	54.06	Kruskal-Wallis (P=0.0003) with Dunn's post-hoc	P=0.0002	P=0.0230	P=0.7571
**Emax (%)**	95.25	10.45	22.85	Kruskal-Wallis (P=0.0002) with Dunn's post-hoc	P=0.0002	P=0.0329	P=0.5087
**EC_{50} [µM]**	2.01		9.63	Mann-Whitney		P=0.0012	
GABBR1	**WT (n=8)**	**p.(I809S) (n=8)**	**1:1 (n=8)**	**Statistics**	**WT& p.(I809S)**	**WT&1:1**	**p.(I809S)&1:1**
**Constitutive activity (%)**	2.17	14.52	8.14	Kruskal-Wallis (P<0.0001) with Dunn's post-hoc	P<0.0001	P=0.0710	P=0.0710
**Emax (%)**	97.84	80.21	83.92	Welch's ANOVA (P=0.0033) with Dunnett's T3 post-hoc	P=0.0508	P=0.0182	P=0.9270
**EC_{50} [µM]**	2.06	0.51	0.99	Kruskal-Wallis (P<0.0001) with Dunn's post-hoc	P<0.0001	P=0.1015	P=0.0776
GABBR1	**WT (n=9)**	**p.(I847V) (n=9)**	**1:1 (n=9)**	**Statistics**	**WT& p.(I847V)**	**WT&1:1**	**p.(I847V)&1:1**
**Constitutive activity (%)**	4.44	14.47	9.20	Welch's ANOVA (P<0.0001) with Dunnett's T3 post-hoc	P<0.0001	P=0.0020	P=0.0009
**Emax (%)**	95.56	77.49	97.69	Welch's ANOVA (P=0.0092) with Dunnett's T3 post-hoc	P=0.0078	P=0.9789	P=0.0480

**EC50 [µM]**	1.76	0.75	1.20	Welch’s ANOVA (P=0.0005) with Dunnett’s T3 post-hoc	P=0.0027	P=0.0778	P=0.0063
GABBR2	**WT (n=8)**	**p.(D165Y) (n=8)**	**1:1 (n=8)**	**Statistics**	**WT& p.(D165Y)**	**WT&1:1**	**p.(D165Y)&1:1**
**Constitutive activity (%)**	1.81	22.4	2.84	ANOVA (P<0.0001) with Tukey’s post-hoc	P<0.0001	P=0.6933	P<0.0001
**Emax (%)**	98.18	53.83	86.92	Welch’s ANOVA (P<0.0001) with Dunnett’s T3 post-hoc	P<0.0001	P=0.2417	P=0.0019
**EC50 [µM]**	2.38	0.83	2.02	Welch’s ANOVA (P=0.0008) with Dunnett’s T3 post-hoc	P=0.0054	P=0.7236	P=0.0007
GABBR2	**WT (n=9)**	**p.(Q430P) (n=6)**	**1:1 (n=9)**	**Statistics**	**WT& p.(Q430P)**	**WT&1:1**	**p.(Q430P)&1:1**
**Constitutive activity (%)**	5.52	-2.29	0.17	Welch’s ANOVA (P=0.0009) with Dunnett’s T3 post-hoc	P=0.0025	P=0.0437	P=0.2148
**Emax (%)**	94.48	0.43	67.55	ANOVA (P<0.0001) with Tukey’s post-hoc	P<0.0001	P<0.0001	P<0.0001
**EC50 [µM]**	0.96		1.32	Unpaired t-test		P=0.0646	
GABBR2	**WT (n=8)**	**p.(M702V) (n=8)**	**1:1 (n=8)**	**Statistics**	**WT& p.(M702V)**	**WT&1:1**	**p.(M702V)&1:1**
**Constitutive activity (%)**	0.70	19.17	21.69	ANOVA (P<0.0001) with Tukey’s post-hoc	P<0.0001	P<0.0001	P=0.3579
**Emax (%)**	99.3	33.36	49.28	Welch’s ANOVA (P<0.0001) with Dunnett’s T3 post-hoc	P<0.0001	P<0.0001	P=0.0039
**EC50 [µM]**	3.01	0.31	0.61	Kruskal-Wallis (P=0.0015) with Dunn’s post-hoc	P=0.0016	P=0.0296	P>0.9999

Supplementary Table S4. IC_{50} of the CGP54626 dose-response curves

+100 µM GABA	**WT (n=7)**	**p.(G531S) (n=8)**	**T-test**
**IC_{50} [µM]**	0.13	145	P<0.0001

Supplementary Figure S1. Evolutionary conservation of variant residues in the GB1 and GB2 subunits

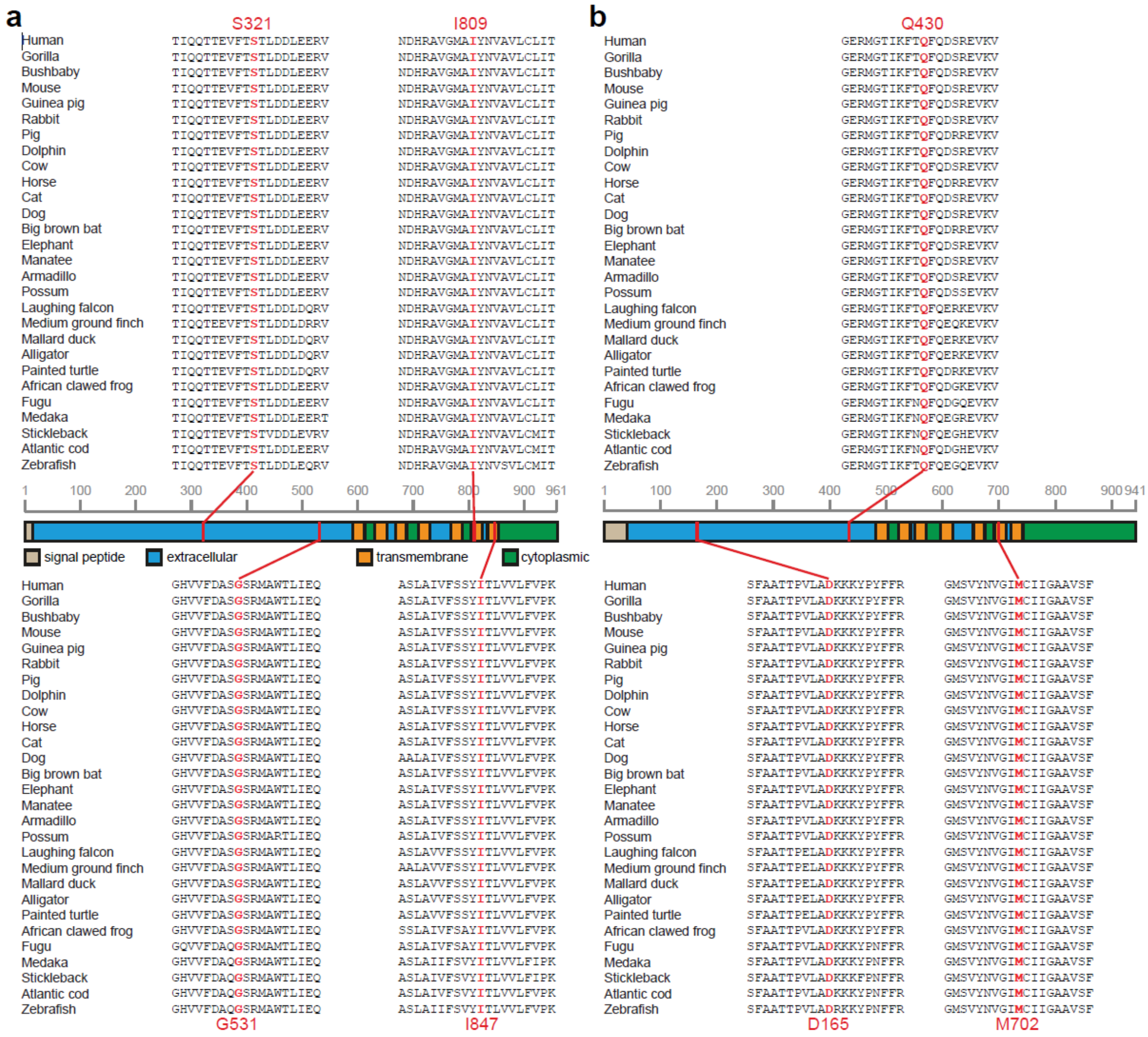

Diagrams show the primary protein structure of GB1 (**a**) and GB2 (**b**) with scale bars above indicating the amino acid position. The signal peptide is depicted in grey, extracellular regions are in blue, the seven transmembrane helices are in yellow and intracellular regions are in green. Multiple sequence alignments of vertebrate GB1 and GB2 homologs were generated with Clustal Omega and trimmed to show 20aa surrounding the residue altered in each variant (indicated in red). The position of each alteration is marked with a red vertical bar in the corresponding diagram.

Supplementary Figure S2. WES results for individual 1, *GABBR1* c.962C>T p.(S321L)

3333 Burnet Avenue, Cincinnati, Ohio 45229

Name: [redacted]
MRN: [redacted]
DOB: [redacted]
Sex: Male
Patient Location: Cincinnati Children's Main Campus Division of Human Genetics
OP ID:
Encounter ID:

Whole Exome Sequencing (Final result)

Result
Positive

Incidental Findings
Opted Out

Clinical History
Clinical history at the time of consultation: 2-year-old with cryptogenic localization-related epilepsy, hypotonia, bicuspid aortic valve and heart murmur, mild global developmental delay, pes planus, bilateral hand and foot differences, macrocephaly, and unique physical characteristics. DNA from the patient and parents was analyzed through this test.

Samples submitted: proband, both parents.

Result Summary
1. Probable Disease Causing Variants or Variants of Unknown Significance Related to the Patient's Phenotype:
Probable disease causing mutation(s) identified in this patient are described in the table below.

Gene (20X)	HGVS	Segregation	Phenotype	Classification
GABBR1 (100.00%)	Chr6(GRCh37):g.29591083G>A NM_001470.3:c.962C>T p.(Ser321Leu)	Proband: Het Father: WT Mother: WT	Association with neurodevelopmental delay and epilepsy (AD)	Likely Pathogenic

Mutation nomenclature is based on the recommendation by American College of Medical Genetics that nucleotide +1 is designated the A of the ATG-translation initiation codon. Abbreviations: AD-autosomal dominant; AR-autosomal recessive; XL-X-Linked; Het-heterozygous; Hom-homozygous; Hem-hemizygous; WT-wild type; NA-not applicable; VUS-Variant of unknown clinical significance.

Pathogenic variants in the *GABBR1* gene are associated with neurological and developmental abnormalities (Rochtus et al. 2020). This gene encodes a subunit of the GABAB receptor expressed in glial cells and neurons. Cerebellar tissue samples from individuals with schizophrenia, bipolar disorder, major depressive disorder, and autism had decreased expression of *GABBR1* when compared with samples from controls (Fatemi et al. 2009; Fatemi et al. 2011). *De novo* monoallelic missense variants in this gene were reported in individuals with developmental delays, intellectual disability, hypotonia, cerebellar atrophy, and epilepsy or seizure disorders (Cediel et al. 2022).

In this family the following variants were identified in GABBR1

This variant, NM_001470.3:c.962C>T (p.Ser321Leu), is a de *novo* missense substitution in exon 8 of 23 that results in the alteration of the codon at amino acid position 321 from Serine to Leucine. In silico predictions for this variant suggest a damaging interpretation. This variant is unreported in dbSNP version 147. This variant is unreported in published literature. This variant has not been previously reported in publicly available population databases, nor has it been reported in association with disease, either in the published literature or ClinVar. Monoallelic de novo variants in *GABBR1* are linked to neurodevelopmental delay and epilepsy (Ceidiel et al. 2022). Based on the available information, this variant is classified as likely pathogenic.

2. Additional Variants of Interest:

The variant(s) identified in this patient are of unknown clinical significance. These variants are described in the table below.

Gene (20X)	HGVS	Segregation	Phenotype	Variant Assessment	Classification
NOTCH1 (100.00%)	Chr9(GRCh37):g.139396827G>A NM_017617.4:c.5281C>T p.(Arg1761Trp)	Proband: Het Father: Het Mother: WT	Adams-Oliver Syndrome 5, (AD) Aortic Valve Disease 1, (AD)	HGMD: Not reported. ClinVar: Not reported. NCBI: rs756191146 Global GnomAD: 0.0008% (2/241316; 0 homo) Max GnomAD (Latino): 0.0029% (1/34264; 0 homo) In-Silico: suggest pathogenic	VUS

Mutation nomenclature is based on the recommendation by American College of Medical Genetics that nucleotide +1 is designated the A of the ATG-translation initiation codon. Abbreviations: AD-autosomal dominant; AR-autosomal recessive; XL-X-Linked; Het-heterozygous; Hom-homozygous; Hem-hemizygous; WT-wild type; NA-not applicable; VUS-Variant of unknown clinical significance.

Clinical Recommendations

Clinical correlation, appropriate clinical evaluation and/or follow-up, and genetic counseling are recommended.

Procedure Methodology

ExomeSeq was performed on genomic DNA using the Human Comprehensive Exome kit from Twist Bioscience to enrich the whole exome. The exome was sequenced using an Illumina sequencing system with paired-end reads at a minimum coverage of 20X of 95% of the target regions. This individual's exome DNA sequences were aligned to the human reference genome (build UCSC hg19) with BWA-mem. Variants were called using GATK, and QC was performed as part of an in-house developed pipeline based on GATK best practices.

Data Analysis Methodology

Variants identified by the GATK-based bioinformatics pipeline were uploaded to the Fabric Genomics Analysis platform, which was used to annotate, analyze, and classify these identified variants. ExomeSeq analysed thousands of genetic variants in this patient, a subset of which were prioritized for interpretation for clinical significance based on the standards and guidelines for the interpretation of sequence variants, recommended by ACMG-AMP (2015). Of these, a final subset of classified variants identified as clinically relevant to the patient's phenotype were then reported. Sanger confirmation was performed on the reported variants unless indicated.

This report only includes variants that met criteria for inclusion in a clinical report. In addition, a subset of variants may be located in regions with inadequate coverage, and therefore an accurate call as to their presence or absence could not be made. ExomeSeq analysis does not include mitochondrial genome sequencing. ExomeSeq does not report identified variants in genes other than those presumed to be related to the patient's phenotype or as specified by ACMG recommendations. Genes that have closely related pseudogenes and regions with high homology, high GC content, polynucleotides and/or nucleotide repeats may not be uniquely and/or optimally captured by this method. Small deletions and insertions of <25 bases are routinely detected by this technology; however, larger deletions, insertions and complex genetic events are not identified using this method. Variants in regulatory regions or other untranslated regions are not detected by this test. Moreover, this test is limited in its use to assess the presence of nucleotide repeat expansions. This test may also not reliably detect mosaicism. Therefore, the absence of identified pathogenic variants does not exclude the possibility of a genetic etiology for the patient's symptoms. Detailed methodologies are available on request.

Procedural References

To obtain additional test details and references related to this analysis, please visit our website

www.cincinnatichildrens.org/diagnosticlabs to access the test spec sheet (PDF) for this assay.

Additional References

Fatemi SH., et al. (2009) Cerebellum. 8(1):64-9. PMID: 19002745
Fatemi SH., et al. (2011) Schizophr Res. 128(1-3):37-43. PMID: 21303731
Rochtus A., et al. (2020) Epilepsia. 61(2):249-258. PMID: 31957018
Cediel ML., et al. (2022) Am J Hum Genet. 2022 Sep 8. pii: S0002-9297(22)00362-7. PMID: 36103875

FDA Statement

This test has not been cleared or approved by the United States Food and Drug Administration. The FDA has determined that such clearance or approval is not necessary. This laboratory is certified under the Clinical Laboratory Improvement Amendments of 1988 ('CLIA') as qualified to perform high complexity clinical laboratory testing. The performance characteristics of this test have been developed and validated by CCHMC Molecular Genetics Laboratory. The interpretation of nucleotide changes is based on our current understanding of the exome. These interpretations may change over time as more information about phenotypes associated with mutations in specific genes becomes available. Exome sequencing was performed at CLIA-certified CCHMC DNA core laboratory.

Electronically signed by [redacted] Date/Time: 10/4/2022 1422

ID:	[redacted]	Type:	Blood
Collected:	6/22/2022 1040	Received:	6/22/2022 1040
Authorized by:	Simpson, Brittany Nicole, M.D.	Verified On:	10/4/2022 1422
Resulting Lab:	CGEN		

Resulting Labs

**CGEN**	CCM GENETICS, 3333 Burnet Ave, Cincinnati OH 45229

Supplementary Figure S3. Sanger chromatograms for individual 2, *GABBR1* c.1591G>A p.(G531S)

Proband

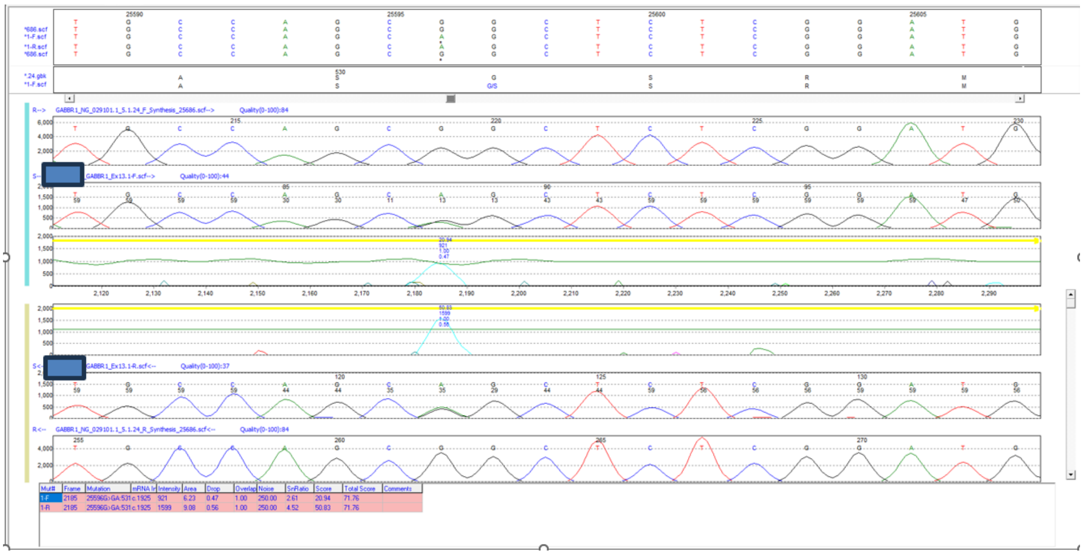

Mother

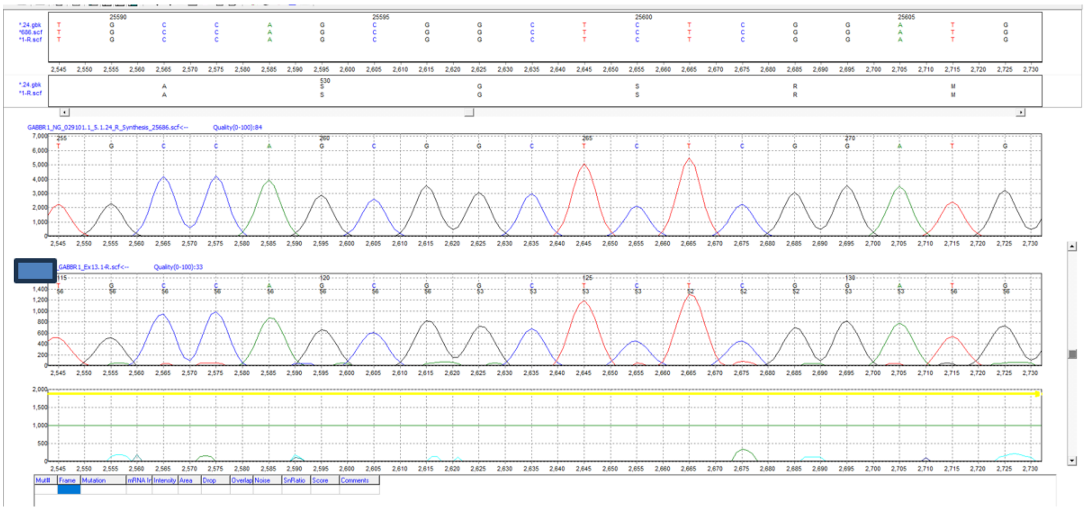

Father

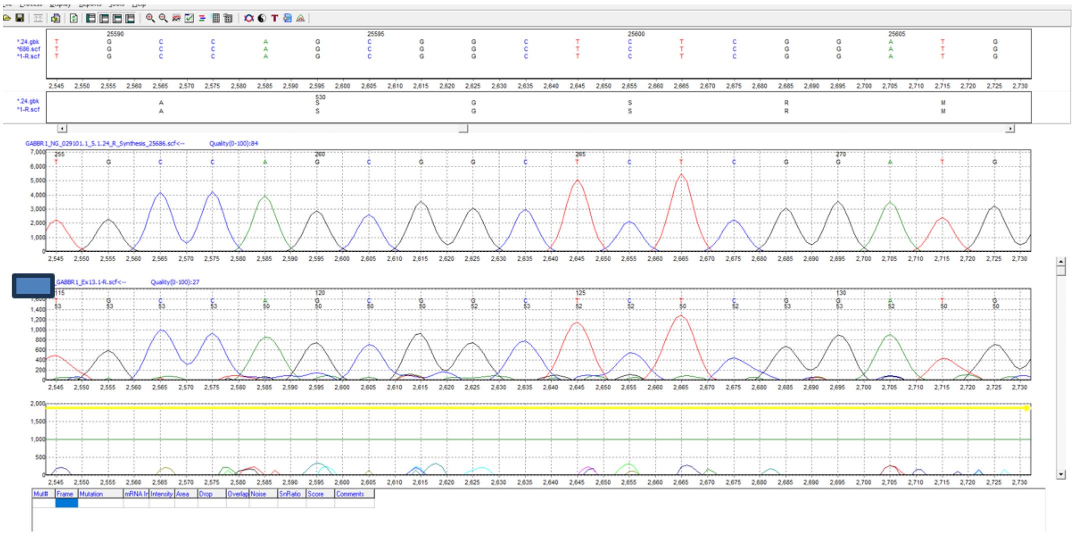

Supplementary Figure S4. WES results for individual 3, *GABBR1* c.2426T>G p.(I809S)

Proband

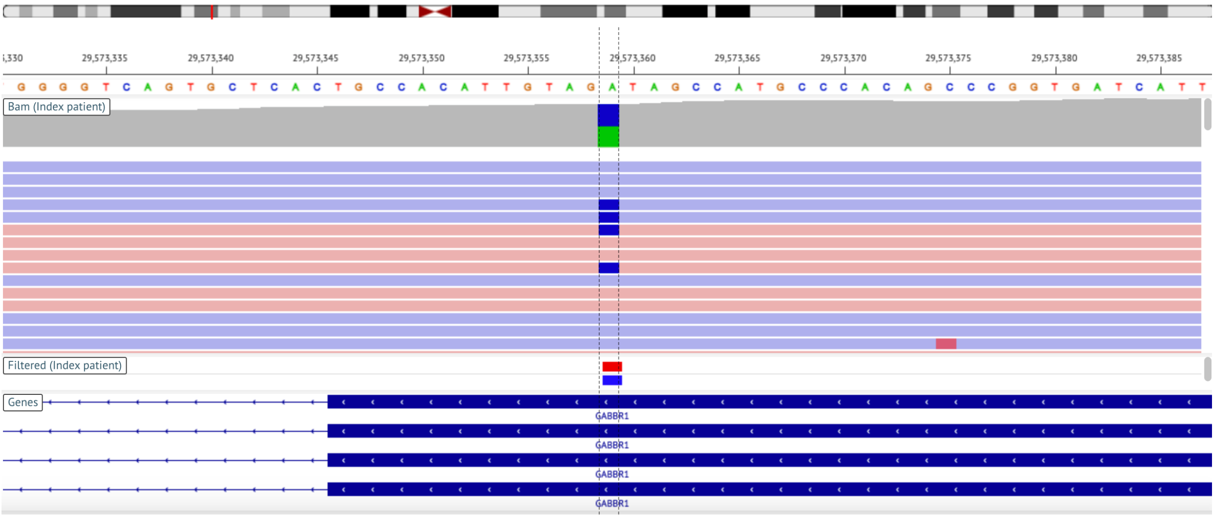

Mother

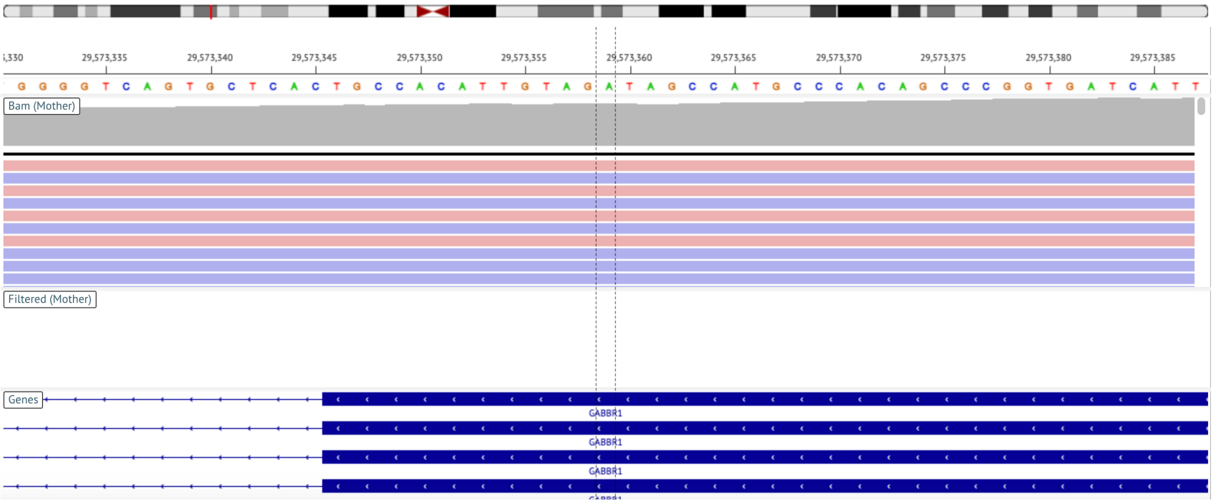

Father

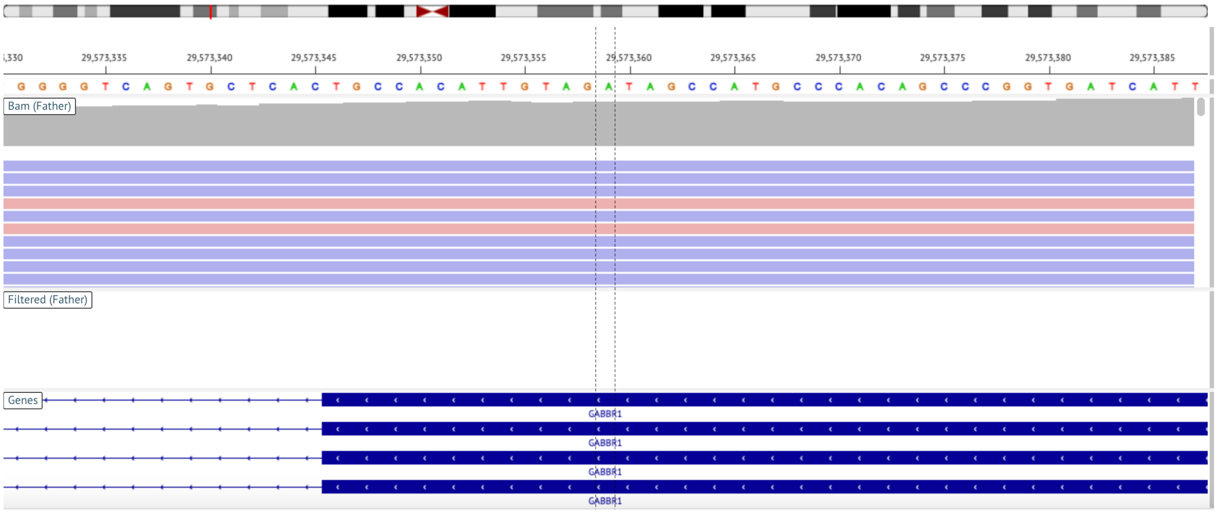

Supplementary Figure S5. WES results for individual 7, *GABBR2* c.2104A>G p.(M702V)

Proband

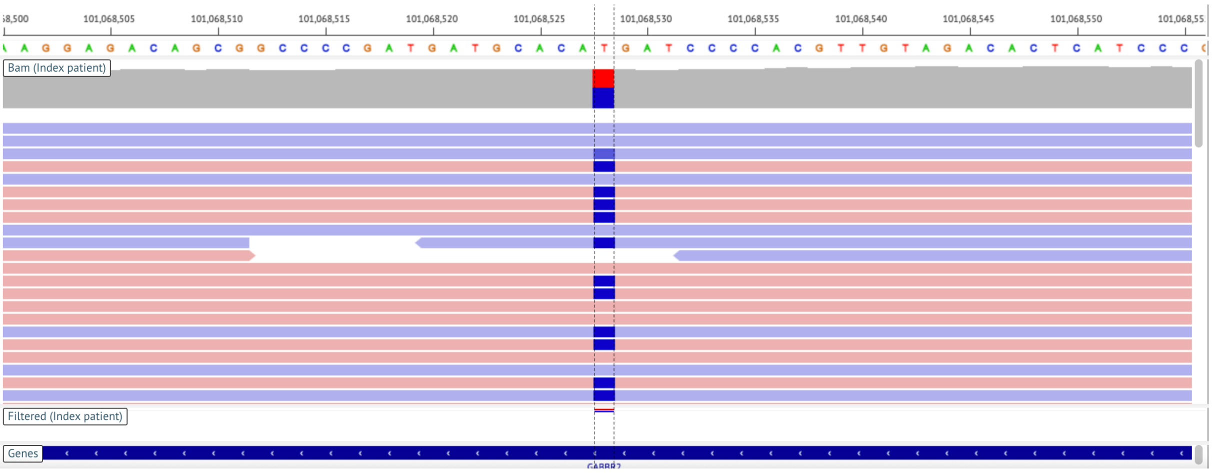

Mother

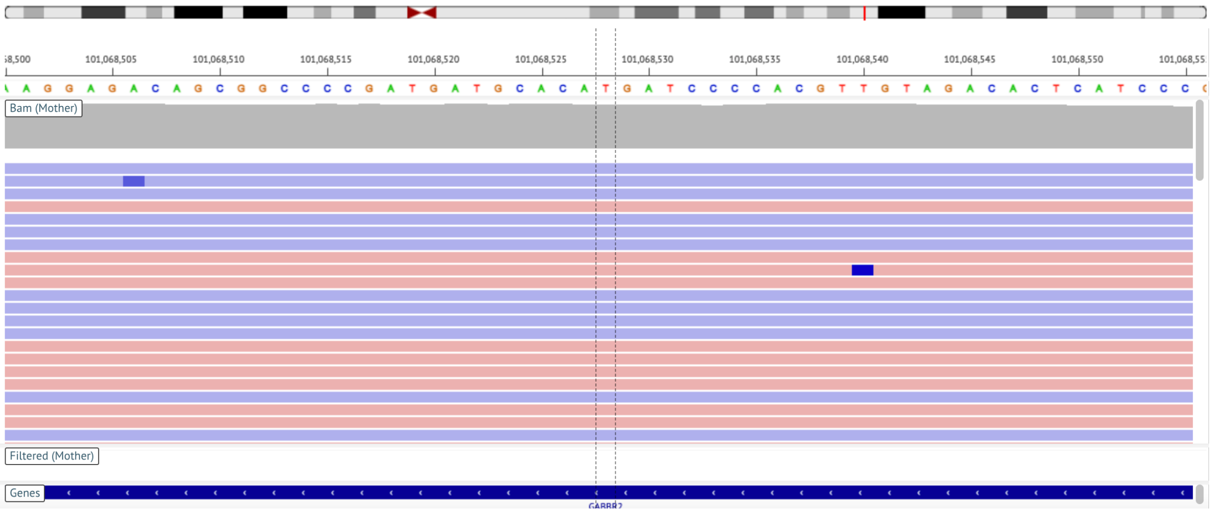

Father

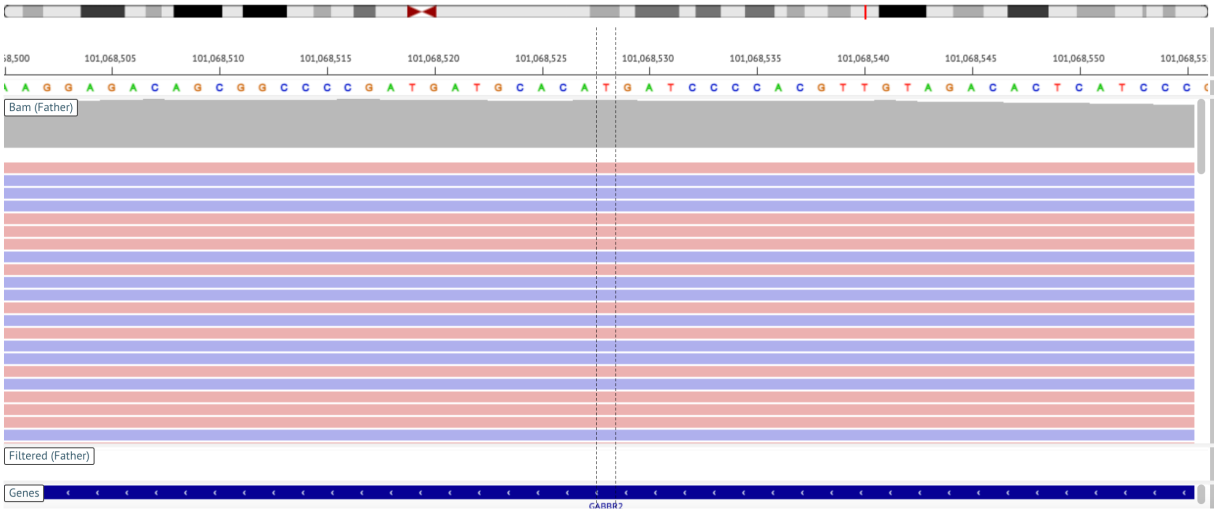

Supplementary Figure S6. Sanger chromatograms for individual 4, *GABBR1* c.2539A>G p.(I847V)

Proband

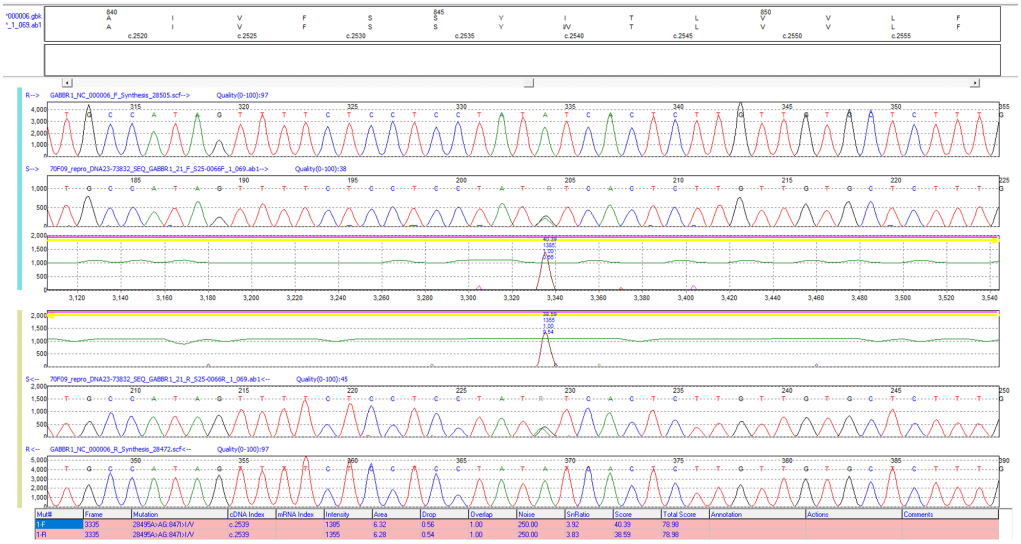

Mother

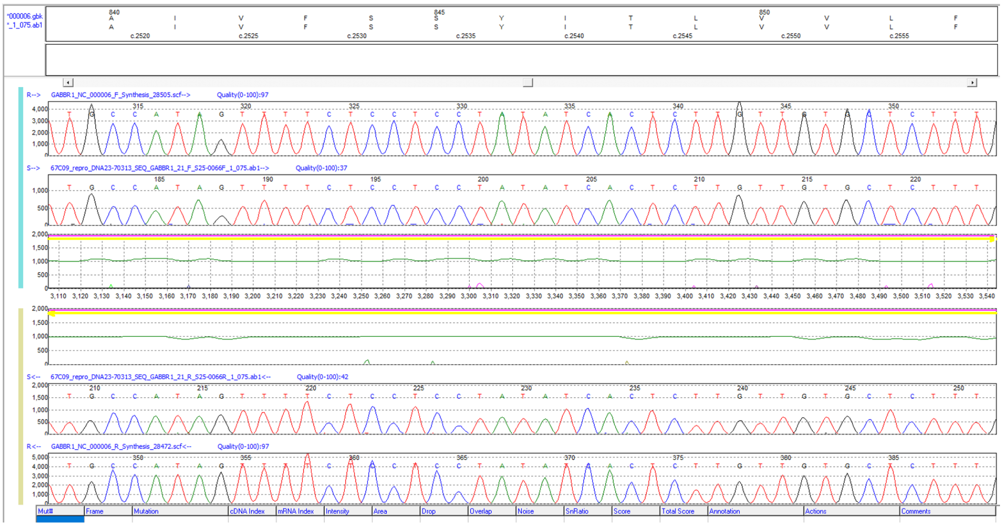

Father

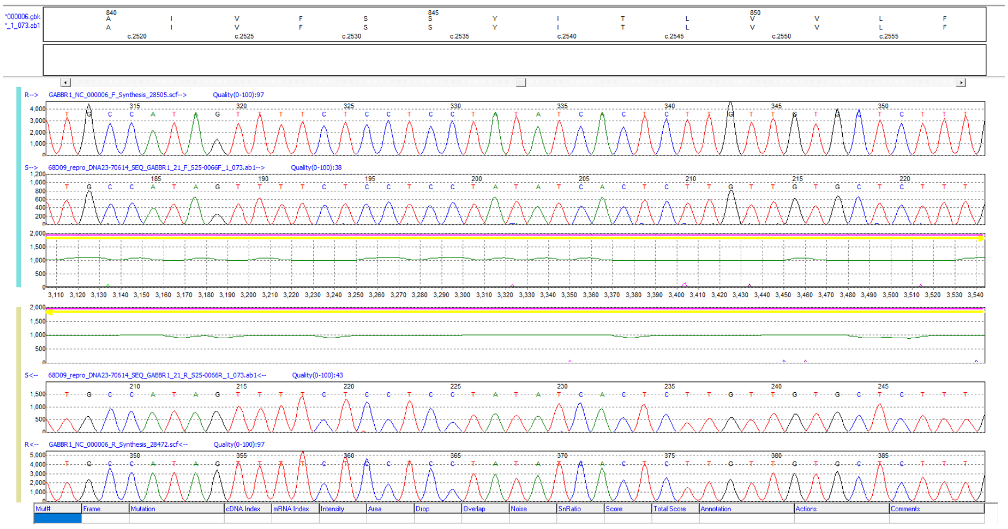

Supplementary Figure S7. WES and ADS results for individual 5, *GABBR2* c.493G>T p.(D165Y)

WES Proband

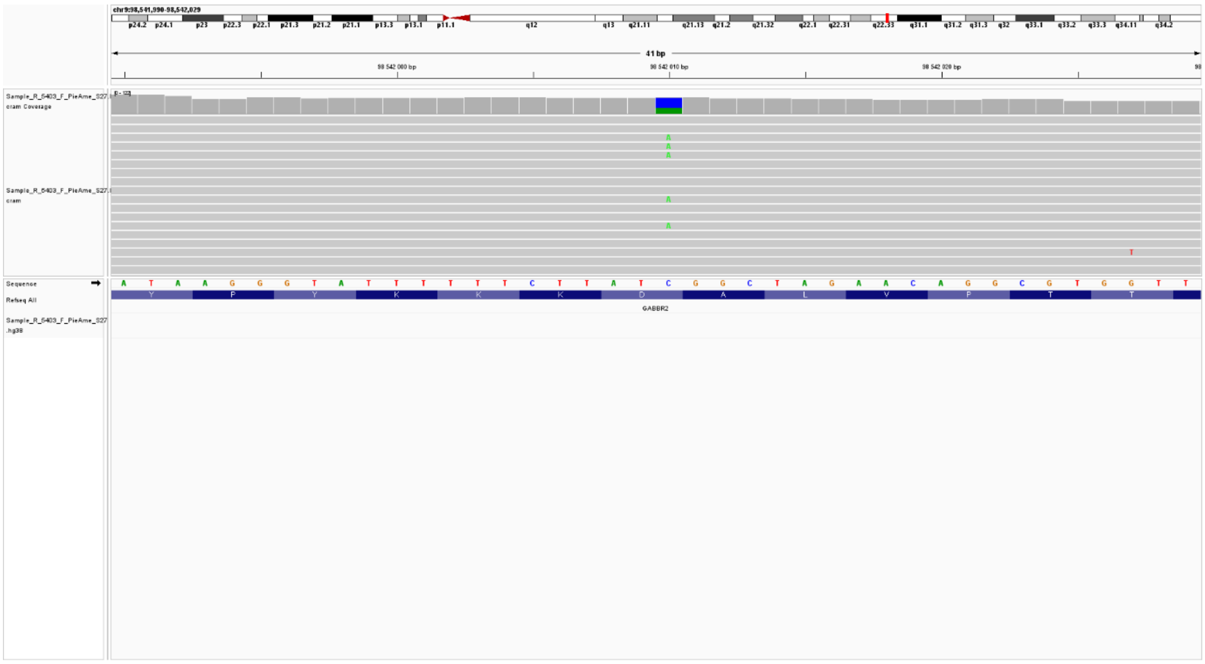

ADS Family

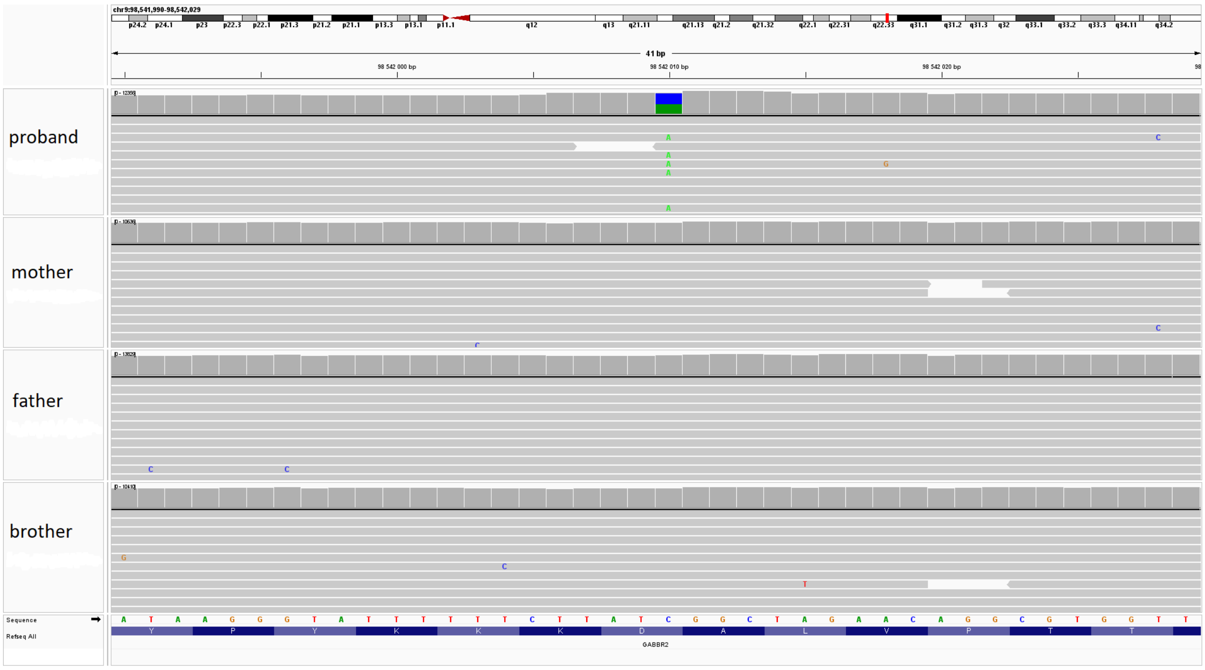

Supplementary Figure S8. Sanger chromatograms for individual 6, *GABBR2* c.1289A>C p.(Q430P)

Forward: top, proband; middle, mother; bottom, father. The heterozygous *GABBR2* c-1289A>C mutation in the proband is in the chromatogram at position 194.

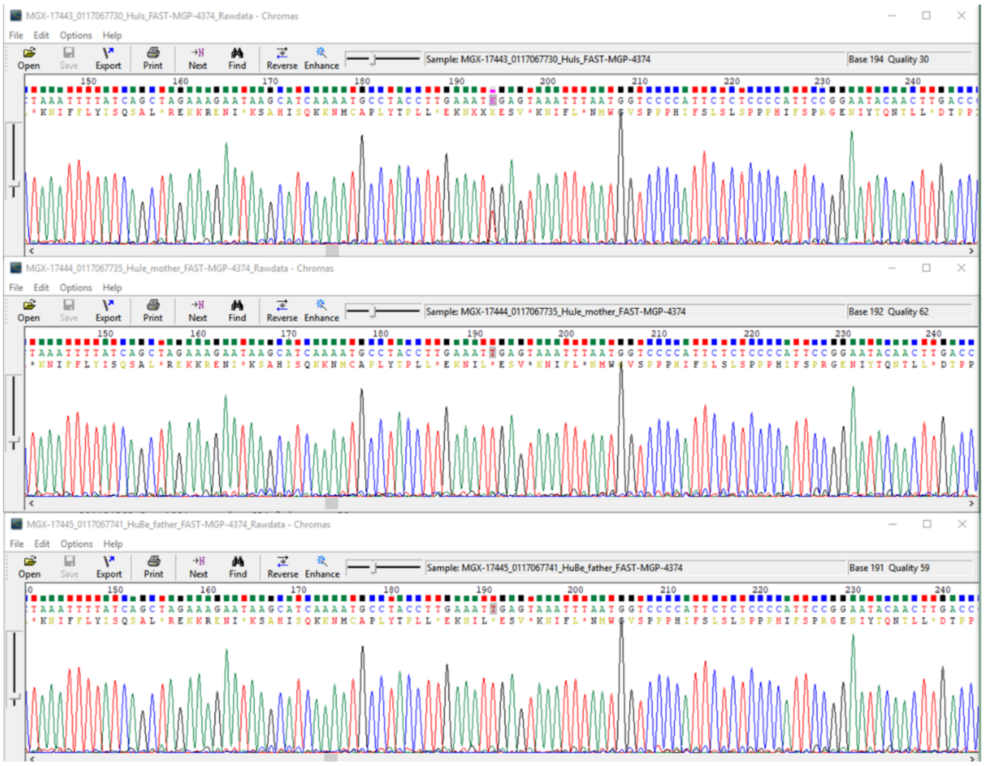

Reverse: top, proband; middle, mother; bottom, father. The heterozygous *GABBR2* c-1289A>C mutation in the proband is in the chromatogram at position 340.

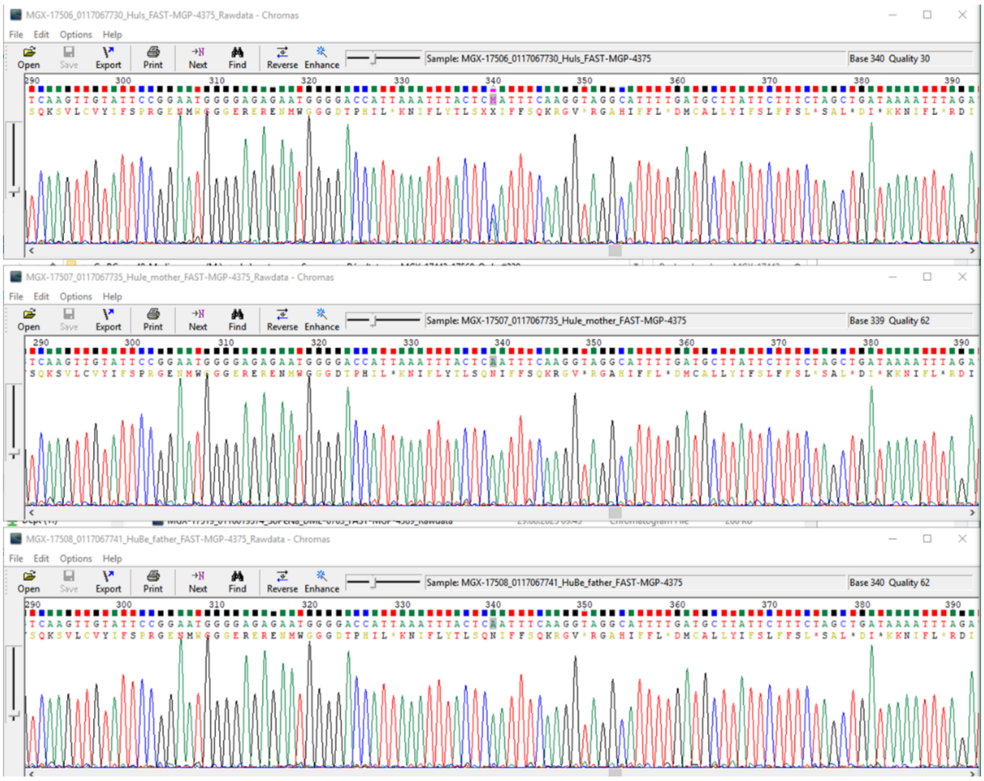

Supplement: Supplementary file 1 — Stawarski et al. NPJ Genomic Medicine_Supplementary Information Revision [file 41525_2026_558_MOESM1_ESM.pdf]
